# Inequalities in the health care system utilization for intrauterine device insertion^1^

**DOI:** 10.1590/1518-8345.7951.4804

**Published:** 2026-03-30

**Authors:** Síntia Nascimento dos Reis, Mery Natali Silva Abreu, Fernanda Penido Matozinhos, Larissa Soares Santos, Ana Paula Lage Guimarães Vallerini, Mariana Santos Felisbino-Mendes

**Affiliations:** 1 Universidade Federal de Minas Gerais, Escola de Enfermagem, Departamento de Enfermagem Materno-Infantil e Saúde Pública, Belo Horizonte, MG, Brazil.; 2 Universidade Federal de Minas Gerais, Escola de Enfermagem, Departamento de Gestão em Saúde, Belo Horizonte, MG, Brazil.; 3 Scholarship holder at the Conselho Nacional de Desenvolvimento Científico e Tecnológico (CNPq), Brazil.; 4 Hospital Sofia Feldman, Belo Horizonte, MG, Brazil.

**Keywords:** Long-Acting Reversible Contraception, Intrauterine Devices, Family Development Planning, Health Inequities, Obstetric Nursing, Delivery of Health Care.

## Abstract

**Objective:**

to investigate the utilization of the Health Care System by women of reproductive age for intrauterine device insertion and to analyze possible inequalities according to sociodemographic characteristics.

**Method:**

cross-sectional study, nested in a prospective cohort, with a random sample of 515 women ≥18 years old, conducted by obstetric nurses at a referral service for intrauterine device insertion. A structured face-to-face questionnaire was applied to obtain information on the use of the healthcare system for intrauterine device insertion. The analysis was based on the Behavioral Model of Health Service Use. Logistic regression and multinomial regression were used to estimate the Odds Ratio of the health care system use according to predisposing factors, race/color, and parity, and facilitating factor, education.

**Results:**

most women are aware of and utilize the system’s health services, especially those with lower levels of education and multiparous women. However, the use of these services to access the intrauterine device is lower, with low access to guidance on reproductive planning and the insertion of the intrauterine device in the services, even lower among those with less education and nulliparous women.

**Conclusion:**

the lower chance of using the health care system to access the intrauterine device among women with less education and nulliparous women reveals social inequalities in access to contraception.

## Highlights

**(1)** Unawareness of IUD insertion at the Primary Health Care Units by registered women. **(2)** Weaknesses in the RAS hinder the equitable use of IUD. **(3)** IUD use reflects inequalities related to parity status. **(4)** Navigation in the Health Care System for using the IUD method. **(5)** A total of 25% of women who access the insertion service do not complete the procedure.

## Introduction

Contraceptive use in Brazil has been concentrated on pills, tubal ligation, and condoms, in that order^1^ ^-^ ^2^. This ranking has been described as obsolete^2^ ^-^ ^3^, as it relies on short-term or surgical methods^2^ ^-^ ^3^. These methods require constant discipline for correct and consistent use^4^, with higher failure rates, or involve risks arising from surgery, as well as high costs or involve surgical risks, as well as high costs.

The use of Long-Acting Reversible Contraception (LARCs), such as the Intrauterine Device (IUD), had a global use rate of approximately 17% in 2020^5^. However, in Brazil, in 2013, the prevalence of IUD use by women of reproductive age was 1.8%^1^, and in 2019, it increased to 4.4%^2^. Thus, the use of the method remains limited in the country compared to the global rate^6^.

Within the scope of the Brazilian Unified Healthcare System (SUS, acronym in Portuguese), the structure of the Health Care System (RAS, acronym in Portuguese) has Primary Health Care (PHC) services as its gateway, where IUD insertion is planned^7^. However, studies point to barriers to access and use of the method^8^ ^-^ ^11^. The most frequently reported facts are that insertion is restricted to doctors^8^ ^-^ ^10^ and the lower availability of the IUD in these services compared to other methods^11^.

Initiatives to increase access to copper IUDs in PHC have previously been described, such as the inclusion of nurses^12^ ^-^ ^13^, as provided for in federal regulations^14^. This strategy has improved access to the method, as has the training of these professionals in some Brazilian contexts^12^ ^-^ ^13^ and in other countries such as Australia^15^, the United States^16^, and India^17^. However, it is still a poorly systematized initiative in Brazil.

Belo Horizonte is among the nine Brazilian capitals that provide all the contraceptive methods stipulated by the Ministry of Health^18^. In the municipality, the IUD can be inserted at the Primary Health Care Units (UBS, acronym in Portuguese) by a specialist doctor, who is not present in 152 of the existing units^19^, which creates a barrier to access to the method. On the other hand, the municipality has a reference outpatient clinic within the RAS, where insertions are performed by trained obstetric nurses, with 6,418 IUD insertions recorded in 2022 and a waiting list of up to five months for them to be carried out^20^. Accessing the women treated at this clinic could help to identify the path of use of health services in the RAS, as well as possible barriers faced by women until they reach IUD insertion.

Theoretical models can support the study of the process of using health services. Andersen’s Behavioral Model of Health Service Utilization^21^ ^)^ is the most widely cited in the literature^22^. In it, access is closely related to the use of health services, which is understood as entry into services and all direct or indirect contact with the service, characterized by the guarantee of care^23^ ^-^ ^24^. It also states that the use of health services is affected by factors from different domains, such as predisposing, facilitating, and need factors^21^ ^,^ ^25^. Predisposing factors refer to sociodemographic and cultural characteristics that make individuals more or less likely to use health services^21^ ^-^ ^22^ ^,^ ^25^, facilitating factors are conditions that make access to health services possible or easier^21^ ^-^ ^22^ ^,^ ^25^, and need factors refer to the perceived and assessed state of health that motivates the use of health services^21^ ^-^ ^22^ ^,^ ^25^.

Studies on contraception and its determinants often report that access to this sexual and reproductive right is still marked by inequalities in both developed countries^26^ and underdeveloped countries^27^ ^-^ ^28^, with greater use of modern contraception among wealthier women with higher education^28^. In Brazil, the prevalence of contraceptive use was lower among black and brown women, as well as among those living in the North and Northeast regions^1^. Another study showed a different sociodemographic profile among users of different types of methods, in which LARCs were more accessed by women with better socioeconomic conditions^2^, corroborating findings in other countries^29^. However, these studies do not address the care pathway of using the RAS to access the methods.

There is a scarcity of studies on the care path that SUS users take within the RAS to access the IUD, as well as the social inequalities in the use of the RAS, previously identified in other care contexts^25^ ^,^ ^30^. Most studies are restricted to investigating the use of contraceptive methods^1^ ^-^ ^2^ ^,^ ^31^. This scientific gap makes it challenging to identify the barriers to IUD use today, contributing to the invisibility of the problem and to contraceptive injustice, since there is a high level of interest in using the method among Brazilian women^32^. These barriers may be due to programmatic and executive problems in the supply of the method, which may reflect the reality of other national and international contexts.

Thus, the first hypothesis of this study is that women encounter difficulties in using the RAS to access the IUD. And the second is that it is more difficult for women with greater social vulnerability to access the IUD, such as lower levels of education, higher parity, and black/brown skin color. Therefore, this study aimed to investigate the use of the Health Care System by women of reproductive age for IUD insertion and to analyze possible inequalities according to sociodemographic characteristics.

## Method

### Type of study

This is a cross-sectional clinical epidemiological study, nested in a prospective cohort, conducted in a reference service for IUD insertion by obstetric nurses in the city of Belo Horizonte, Minas Gerais.

### Population, sample, inclusion and exclusion criteria

For the prospective cohort, a representative simple random sample was calculated for the municipality based on the number of IUD insertion consultations carried out by the service in 2022, stratified by type of IUD (copper and hormonal) and time of insertion (postpartum and interval). The parameters of discontinuation were used as the primary outcome, and low education as the exposure. The calculation was carried out for a significance level of 5%, 80% power, a discontinuation rate of 46.2% in the high education group (not exposed), and an Odds Ratio of 1.71^33^, totaling 444 women with IUD insertion. This figure was increased by 10% considering possible loss to follow-up, resulting in a total of 489 women having an IUD inserted after the consultation. The OpenEpi program version 3.01 was used to calculate the study sample.

Eligible participants were women aged 18 years or older, living in the municipality of Belo Horizonte, nulliparous and multiparous, who met the eligibility criteria for using the contraceptive method offered^34^.

Participating women who were already using the IUD, with suspected or confirmed pregnancy, or cognitive impairment, and those who had the IUD inserted in the postpartum period (n=30) were excluded, given the differentiated access to this modality, mainly due to prior confirmation of the absence of pregnancy and the possibility of insertion during hospitalization. Specific criteria were also applied according to the type of IUD. For copper IUDs, women with allergies to copper, cervical or endometrial cancer, uterine anomalies, cervical stenosis, immunosuppression, use of anticoagulants, or coagulation disorders^34^ were excluded. In the case of hormonal IUDs, those whose use was indicated for the treatment of comorbidities such as abnormal uterine bleeding, endometriosis, adenomyosis, leiomyomatosis, or endometrial hyperplasia were excluded^35^.

Thus, of the total of 545 women who answered the baseline questionnaire, after applying the exclusion criteria, 515 women were included in the study, regardless of whether they had adopted the contraceptive method.

### Variables of interest 

The outcome variables in this study refer to the use of RAS services for IUD insertion, as proposed by Andersen^24^: knowledge of the UBS of reference; registration at the UBS; attendance at the UBS in the past 12 months; discussed reproductive planning with a professional; consultation with a professional to choose the IUD; knowledge of whether the UBS inserts IUDs; thought about giving up; attempt to insert the IUD at the UBS (categorized as yes and no); number of attempts to insert the IUD at health services before attendance; number of professionals consulted for IUD insertion (none, one, two or more); which professional consulted for IUD insertion (nurse, doctor, other); length of time waiting for IUD insertion (<1, 1-3, 4-6, 7 months or more); and who informed them about IUD insertion at the hospital (nurse, doctor, maternity admission, friend/relative, internet; referral from UBS service, other).

The selection of exposure variables was also guided by the Behavioral Model of Health Services Utilization^24^. Therefore, individual sociodemographic characteristics were considered predisposing factors^36^ and included race/color (white, black, and brown) and parity (none, one to two, and three or more). Education (0-12 and 13 or more years of study) was included as a facilitating factor.

Other covariates relating to sociodemographic and reproductive characteristics were used to describe the study population and their contraceptive needs: age group (18-24, 25-29, 30-34, and 35 years or older); marital status (single/widowed or divorced, married/stable union); sexual partnership (yes, no); paid work (yes, no); occurrence of previous pregnancy (yes, no); pregnancy was planned (yes and no); wishes to have more children (yes, no); currently uses some method (yes, no); age at first pregnancy (under 18, 18-24, and 25 or over); level of satisfaction with current method (indifferent, satisfied/very satisfied, unsatisfied/very unsatisfied). None of the women answered some variables, and those who declared themselves to be yellow (n=10), indigenous (n=1), or did not inform their race/color (n=7) were excluded from the analysis due to the very low and unrepresentative number of categories. 

### Data collection 

The cohort’s baseline data collection took place between September 2023 and August 2024 at a philanthropic institution that provides its services entirely through the SUS. The service is recognized as a reference in the work of obstetric nurses, especially in the insertion of the IUD in the context of reproductive health care.

For data collection, a questionnaire was developed based on questions from consolidated instruments, such as the 2006 Brazilian National Survey of Demography and Health (PNDS, acronym in Portuguese) and the 2013 and 2019 editions of the National Health Survey (PNS, acronym in Portuguese). The questionnaire was tested in a pilot study with seven women, which allowed for adjustments to improve clarity and coherence, making the instrument suitable for use in data collection. In the end, the questionnaires were structured and organized into 12 thematic modules: identification; inclusion and exclusion criteria; contact information for follow-up; decision-making process for IUD use; trajectory in the Health Care System (RAS, acronym in Portuguese); knowledge about the method; sociodemographic characteristics; reproductive history; and current sexual and reproductive health.

The interviews were conducted using a questionnaire, face-to-face, on the day scheduled for the IUD insertion appointment, while the users were waiting to be seen. The participants were selected by random sampling, based on the day’s appointment schedule, considering the availability of the interviewers and the scheduled appointments. Once the questionnaire and consultation had been completed, the method insertion was confirmed. Data was collected using the Research Electronic Data Capture (REDCap) software.

The data collection team was previously trained, receiving both theoretical and practical instruction, including training on IUDs. It consisted of undergraduate nursing students, nurses, obstetric nurses, and students pursuing master’s and doctoral degrees in nursing. The training involved a presentation of the research project, a thematic workshop on IUDs, a detailed presentation of the data collection manual, an explanation of the questionnaire, and practical training with simulated interviews using the REDCap software on mobile devices (cell phones and tablets). During data collection, monthly monitoring meetings were held with the team to discuss operational difficulties and implement continuous improvements in the quality of the data obtained. This process aimed to ensure that the questionnaire was completed correctly and that the approach to the participants was standardized.

### Data analysis

First, the study population was described according to sociodemographic and reproductive characteristics, using absolute (n) and relative (%) frequencies. Next, the same descriptive analysis was carried out for the groups of women who inserted and did not insert the IUD after the evaluation in the consultation. Subsequently, the proportion of RAS utilization variables was calculated for the total number of women, and for the groups of women who inserted and those who could not insert the IUD.

In this study, sociodemographic characteristics such as race/color and parity were considered predisposing factors, while education was considered a facilitating or enabling factor for using the RAS to obtain the IUD as a contraceptive method. Thus, was analyzed whether the sociodemographic characteristics of the women who achieved IUD insertion (self-reported skin color/race, parity, and education) were associated with the variables for using RAS. At this stage, Pearson’s chi-square test and Fisher’s exact test were used to assess statistical differences between the proportions. Next, the unadjusted Odds Ratio (ORna) was estimated only for the factors that showed a p <0.2 in the descriptive analysis. Logistic regression was used for binary utilization variables and multinomial logistics for those with more than two responses. All the analyses were carried out using STATA software, version 16.1.

### Ethical aspects

The study was conducted in accordance with national and international ethical guidelines and approved by the Research Ethics Committee of the Federal University of Minas Gerais (opinion no. 6.074.135; CAAE: 68438523.60000.5149) and the Sofia Feldman Hospital (opinion no. 6.091.977; CAAE: 68438523.6.3001.5132), on May 23rd and 31st, 2023, respectively. All the participants signed the Free and Informed Consent Form before the interviews, which is attached to this submission.

## Results

The sociodemographic and reproductive characteristics of the women studied, in total and according to IUD insertion status, are shown in Table 1. The majority were young, aged between 18-24 (37.5%) and 25-29 (27.9%), self-declared as black or brown (73.5%); were single, widowed, or divorced (71.7%); and had a current sexual partner (77.1%). Additionally, most of the women had completed up to 12 years of education and were employed. 

As for reproductive history, of the 507 women who answered about previous pregnancies, 59.4% said they had been pregnant. Of these, the age at first pregnancy was between 18 and 25 for 59.2%, and the majority had not planned the pregnancy (60.4%). Of all the women interviewed, most did not want to get pregnant again and were using some form of contraception at the time of the consultation, mainly condoms and the pill. They were also satisfied/very satisfied with their current method (67.1%). When asked about changing their current method for the IUD, the majority reported looking for more safety (40.6%), followed by practicality (18.8%) and due to side effects (15.5%).

According to Table 1, there was no difference in sociodemographic and reproductive characteristics between the groups who did or did not achieve insertion on the date of the appointment. It should be noted that, for every four women who accessed the referral service, one was unsuccessful in inserting the IUD, despite being scheduled for the procedure (25.4%; n=130). The main reasons for not inserting the IUD were the impossibility of ruling out pregnancy (37.0%) and the desire for another type of IUD not available at the service (24.0%). 

**Table 1:** Proportion of sociodemographic and reproductive characteristics of women seen at the reproductive planning clinic according to intrauterine device insertion status (n = 515). Belo Horizonte, MG, Brazil, 2024

Sociodemographic and reproductive characteristics	Total	Situation IUD insertion*				P-value^||^
		Did not insert		Inserted		
	n (%)^†^	N^‡^	%^§^	N^‡^	%^§^	
**Age (years) (n^¶^=509)**						0.722
18-24	191 (37.5)	54	41.5	137	36.1	
25-29	142 (27.9)	35	27.0	107	28.2	
30-34	93 (18.3)	21	16.1	72	19.0	
35 or more	83 (16.3)	20	15.4	63	16.7	
**Self-declared color (n^¶^=491)**						0.836
White	130 (26.4)	33	25.8	97	26.7	
Black and brown	361 (73.6)	95	74.2	266	73.3	
**Marital status (n^¶^=509)**						0.531
Single, widowed, or divorced	365 (71.7)	96	73.9	269	71.0	
Married or in a stable union	144 (28.3)	34	26.1	110	29.0	
**Education (years of study) (n^¶^=509)**						0.205
0 to 12	317 (62.3)	87	66.9	230	60.7	
13 or more	192 (37.7)	43	33.1	149	39.3
**Currently working (n^¶^=509)**						0.739
Yes	393 (77.2)	99	76.1	294	77.6	
**Previous pregnancy (n^¶^=507)**						0.261
Yes	301 (59.4)	82	63.6	219	57.9	
**Number of pregnancies (n^¶^=507)**						0.308
0	206 (40.6)	47	36.4	159	42.0	
1	168 (33.1)	51	39.5	117	30.9	
2	88 (17.4)	22	17.0	66	17.5	
3 or more	45 (8.9)	9	7.0	36	9.5	
**Planned pregnancy (n^¶^=303)**						0.358
No	183 (60.4)	53	60.4	130	58.8	
**Age at first pregnancy (years) (n^¶^=302)**						0.633
<18	66 (21.9)	15	18.3	51	23.2	
18-25	179 (59.3)	50	61.0	129	58.6	
26 or more	57 (18.8)	17	20.7	40	18.2	
**No desire to have more children (n^¶^=508)**						0.440
No	296 (58.3)	72	55.4	224	59.3	
Currently using CM** (n^¶^=502)						0.080
Yes	356 (71.0)	83	64.8	273	73.0	
**Current method (n^¶^=356)**						0.094
Condoms	124 (34.9)	25	30.1	99	36.3	
Combined pill or mini pill	108 (30.3)	25	30.1	83	30.4	
Injectable	57 (16.0)	47	12.1	10	17.2	
IUD* copper and hormonal	52 (14.6)	16	19.3	36	13.2	
Others^††^	15 (4.2)	7	8.4	8	2.9	
Satisfaction with current CM** (n^¶^=356)						0.003
Indifferent	49 (13.8)	10	12.1	39	14.3	
Satisfied/very satisfied	239 (67.1)	49	59.0	190	69.6	
Unsatisfied/very unsatisfied	68 (19.1)	24	28.9	44	16.1	
**Current sexual partner (n^¶^=507)**						0.542
Yes	391 (77.1)	102	79.1	289	76.4	

*IUD = Intrauterine Device; ^†^n(%) = Absolute frequency and percentage; ^‡^N = Total sample; ^§^% = Percentage; ^||^p-value = Pearson’s chi-square test; ^¶^n = Total number of people who answered the question; **CM = Contraceptive method; ^††^Other = Natural methods, emergency pill and lactational amenorrhea method

According to Table 2, regarding the use of HCN health services up to the day of the appointment at the referral service, it was observed that most women were aware of the referral UBS, were registered, and had sought care at the UBS within the last year. However, only 34.6% had attended a Reproductive Planning (RP) appointment before choosing the IUD; 74.4% had tried to have the device inserted at the UBS, and only 20.9% were aware of this possibility. Of those who consulted a health professional to choose the IUD, only 32.2% consulted a nurse.

Additionally, according to Table 2, access to the outpatient clinic for IUD insertion was primarily through referrals from friends, relatives, and other individuals (83.3%), with a relatively low proportion of referrals from professionals in RAS services. Regarding waiting times, 57.8% of women waited between 1 and 3 months, while 30.6% waited 4 months or more. There were no significant differences in the use of RAS in relation to the insertion of IUDs.

Next, the use of RAS was analysed by skin color/race, education, and parity for women who achieved IUD insertion (n=363). Table 3 shows that there were no differences in the likelihood of using RAS based on skin color/race.

The results in Table 4 show the use of the RAS according to education, in which it can be seen that women with a higher level of education, compared to those with a lower level of education, made greater use of the services of the UBS in the last year (OR=3.21; 95% CI: 1.84-5.60), but were less likely to be seen at a specific appointment for choosing the IUD (OR=0.61; 95% CI: 0.40-0.93).

**Table 2:** Proportion of RAS utilization* by women seen at the reproductive planning outpatient clinic to access the intrauterine device at the referral service according to the insertion status of the intrauterine device (n = 515). Belo Horizonte, MG, Brazil, 2024

RAS Utilization Variables*	Total	Situation IUD insertion^†^				P-value^¶^
		Not Inserted		Inserted		
	n (%)^‡^	N^§^	%^||^	N^§^	%^||^	
Knows reference UBS** (n^††^=509)						0.919
Yes	449 (88.2)	115	88.5	334	88.1	
UBS registration** (n^††^=450)						0.426
Yes	431 (95.8)	112	97.4	319	95.2	
Attended UBS** in the last year (n^††^=423)						0.924
Yes	331 (78.2)	88	78.6	243	78.1	
**Discussed RP^‡‡^ with a professional (n^††^=508)**						0.060
Yes	175 (34.6)	36	27.7	139	36.7	
**Consultation with a professional to choose an IUD^†^ (n^††^=509)**						0.955
Yes	320 (62.9)	82	63.1	238	62.8	
**Professional consultation (n^††^=246)**						0.612
Physician/doctor	167 (67.8)	40	63.5	127	69.4	
Nurse	79 (32.2)	23	36.5	56	30.6	
**Number of professionals consulted (n^††^=506)**						0.216
None	260 (51.4)	66	51.2	194	51.5	
One	187 (36.9)	53	41.1	134	35.5	
Two or more	59 (11.7)	10	7.7	49	13.0	
Knows about insertion in UBS** (n^††^=450)						0.995
Yes	94 (20.9)	24	20.9	70	21.0	
Attempted insertion into UBS** (n^††^=450)						0.910
Yes	335 (74.4)	98	21.7	237	52.7	
**Source of IUD information^†^ (n^††^=515)**						0,939
Physician/doctor	41 (8.1)	11	8.5	30	7.9	
Nurse	44 (8.6)	12	9.3	32	8.4	
Others^§§^	424 (83.3)	107	82.3	317	83.6	
**Waiting time for IUD insertion^†^ (in months) (n^††^=509)**						0.040
<1	59 (11.6)	11	8.5	48	12.6	
1-3	294 (57.8)	83	63.9	211	55.7	
4-6	79 (15.5)	12	9.2	67	17.7	
7 or more	77 (15.1)	24	18.5	53	14.0	
**Number of IUD insertion attempts^†^ (n^††^=507)**						0.888
None	215 (42.4)	53	40.8	162	43.0	
One	202 (39.8)	54	41.5	148	39.3	
Two or more	90 (17.8)	23	17.7	67	17.8	
**Thought about giving up (n^††^=509)**						0.281
Yes	112 (22.0)	33	25.4	79	20.8	

*RAS = Health Care System; ^†^IUD = Intrauterine Device; ^‡^n(%) = Absolute frequency and percentage; ^§^N = Total sample; ^||^% = Percentage; ^¶^p-value = Pearson’s chi-squared test; **UBS = Primary Health Care Units; ^††^n = Total number of people who answered the question; ^‡‡^RP = Reproductive Planning; ^§§^Others = Friend/Parent/Concierge, secretary and teacher

**Table 3:** Proportion and Odds Ratio (OR) of RAS utilization* by women who had an intrauterine device inserted after attending the reproductive planning clinic to access the contraceptive method at the referral service according to race/color (n = 363). Belo Horizonte, MG, Brazil, 2024

RAS Utilization Variables*	Race/Color		valor-p^‡^	Race/Color	
	White	Black/Brown		White	Black/Brown
	n (%)^†^	n (%)^†^		ORna^§^ (CI95%)^||^	
Knows reference UBS^¶^ (n**=363)			0.357		
Yes	83 (85.6)	237 (89.1)			
Registration at UBS^¶^ (n**=321)			0.275		
Yes	77 (92.8)	228 (95.8)			
Attended UBS^¶^ in the last year (n**=298)			0.234		
Yes	56 (73.7)	178 (80.2)			
Discussed RP^††^ with a professional (n**=363)			0.719			
Yes	37 (38.1)	96 (36.1)		Ref.^‡‡^	1.09 (0.68-1.76)
Consultation with a professional to choose an IUD^§§^ (n**=363)			0.820		
Yes	37 (38.1)	98 (36.8)			
Professional consultation (n**=108)			0.683		
Physician/doctor	13 (52.0)	47 (56.6)			
Nurse	12 (48.0)	36 (43.4)		Ref.^‡‡^	1.21 (0.49-2.95)
Number of professionals consulted (n**=363)			0.623		
None	49 (50.5)	137 (51.9)			
One	38 (39.2)	93 (35.0)		Ref.^‡‡^	1.14 (0.69-1.88)
Two or more	10 (10.3)	36 (13.5)		Ref.^‡‡^	0.78 (0.36-1.68)
Knows the insertion in the UBS^¶^ (n**=321)			0.334		
Yes	14 (16.9)	52 (21.9)			
Attempting to join the UBS^¶^ (n**=321)			0.485			
Yes	26 (31.3)	65 (27.3)		Ref.^‡‡^	0.82 (0.48-1.42)
Source of information on IUDs^§§^ (n**=363)			0.619			
Physician/doctor	21 (7.9)	6 (6.2)		
Nurse	20 (7.5)	10 (10.3)		Ref.^‡‡^	1.75 (0.54-5.71)
Others^||||^	225 (84.6)	81 (83.5)		Ref.^‡‡^	1.26 (0.49-3.23)
Waiting time for IUD insertion^§§^ (in months) (n**=363)			0.232			
<1	13 (13.4)	33 (12.4)			
1-3	52 (53.6)	153 (57.5)			
4-6	22 (22.7)	39 (14.1)			
7 or more	10 (19.6)	41 (80.4)			
Number of IUD insertion attempts^§§^ (n**=361)			0.972			
None	42 (43.7)	114 (43.1)			
One	38 (39.6)	104 (39.2)			
Two or more	16 (16.7)	47 (17.7)			
Thought about giving up (n**=363)			0.124			
Yes	25 (25.8)	49 (18.4)		Ref.^‡‡^	1.54 (0.89-2.67)

*RAS = Health Care System; ^†^n (%) = Absolute frequency and percentage; ^‡^Pearson’s chi-square test; ^§^ORna = Unadjusted Odds Ratio; ^||^IC95% = 95% Confidence Interval; ^¶^UBS= Primary Health Care Units; **n = Total number of people who answered the question; ^††^RP = Reproductive Planning; ^‡‡^Ref. = Reference category; ^§§^IUD = Intrauterine Device; ^||||^Friend/Parent/Concierge, secretary and teacher

**Table 4:** Proportion and Odds Ratio (OR) of RAS utilization* by women who had an intrauterine device inserted after attending the reproductive planning clinic to access the contraceptive method at the referral service according to schooling (n = 379). Belo Horizonte, MG, Brazil, 2024

RAS Utilization Variables*	Education (in years)		valor-p^‡^	Education (in years)	
	0 to 12	13 or more		0 to 12	13 or more
	n (%)^†^	n (%)^†^		ORna^§^ (CI95%)^||^	
Knows reference UBS^¶^ (n**=379)			0,282			
Yes	206 (89.6)	128 (86.0)				
Registration at the UBS^¶^ (n**=335)			0.333			
Yes	198 (96.1)	121 (93.8)			
Attended UBS^¶^ in the last year (n**=311)			<0.0001^††^			
Yes	78 (65.6)	165 (85.9)		Ref.^‡‡^	3.21 (1.84-5.60)^§§^
Discussed RP^||||^ with professional (n**=378)			0.073			
Yes	63 (42.3)	76 (33.2)		Ref.^‡‡^	0.68 (0.44-1.04)
Consultation with a professional to choose an IUD^¶¶^ (n**=379)			0.021^††^			
Yes	66 (44.3)	75 (32.6)		Ref.^‡‡^	0.61 (0.40-0.93)^§§^
Professional consulted (n**=113)			0.342			
Physician/doctor	24 (60.0)	37 (50.7)		Ref.^‡‡^	
Nurse	16 (40.0)	36 (49.3)			1.46 (0.67-3.19)
Number of professionals consulted (n**=379)			0.090			
None	66 (44.3)	128 (55.7)		Ref.^‡‡^	
One	59 (39.6)	75 (32.6)			0.66 (0.42-1.03)
Two or more	24 (16.1)	27 (11.7)			0.58 (0.31-1.08)
Knows the insertion in the UBS^¶^ (n**=335)			0.414			
Yes	24 (18.6)	46 (22.3)			
Attempt to join the UBS^¶^ (n**=335)			0.096			
Yes	31 (24.0)	67 (32.52)		Ref.^‡‡^	1.52 (0.93-2.51)
Source of information on IUDs (n**=379)			0.783			
Physician/doctor	11 (7.4)	19 (8.3)		Ref.^‡‡^	
Nurse	11 (7.4)	21 (9.1)			1.10 (0.39-3.13)
Others***	127 (85.2)	190 (82.6)			0.87 (0.40-1.88)
Waiting time for IUD insertion^¶¶^ (in months) (n**=379)			0.015^††^			
<1	24 (16.1)	24 (10.4)		Ref.^‡‡^	
1-3	74 (49.7)	137 (59.6)			1.85 (0.98-3.48)
4-6	35 (23.5)	32 (13.9)			0.91 (0.44-1.92)
7 or more	16 (10.7)	37 (16.1)			2.31 (1.02-5.22)^§§^
Number of IUD insertion attempts^¶¶^ (n**=377)			0.043^††^		
None	61 (41.2)	101 (44.1)		Ref.^‡‡^	
One	68 (45.9)	80 (34.9)			0.71 (0.45-1.12)
Two or more	19 (12.8)	48 (21.0)			1.56 (0.82-2.83)
Thought about giving up (n**=379)			0.988		
Yes	48 (28.9)	31 (20.8)			

*RAS = Health Care System; ^†^n (%) = Absolute and percentage frequency; ^‡^Pearson’s chi-square test; ^§^ORna = Unadjusted Odds Ratio; ^||^95%CI = 95% Confidence Interval; ^¶^UBS = Primary Health Care Units; **n = Total number of people who answered the question; ^††^Statistically significant p-value (p<0.05); ^‡‡^Ref. = Reference category; ^§§^Odds ratios with statistical significance, identified by the 95%CI which does not include the null value (OR = 1); ^||||^RP = Reproductive Planning; ^¶¶^IUD = Intrauterine Device; ***Friend/Parents/Concierge, secretary, and teacher

Regarding the analysis of RAS utilization according to parity, Table 5 shows that multiparous women were more likely to know the UBS of reference (OR=2.83; 95%CI 1.48-5.42) and to know about IUD insertion at the UBS (OR=3.14; 95%CI 1.66-5.91) compared to nulliparous women. However, these women were less likely to wait 1-3 months for IUD insertion (OR=0.42; 95% CI 0.21-0.83) compared to nulliparous women. In addition, they were more likely to have had more insertion attempts (2 or more attempts: OR=2.15; 95% CI 1.17-3.96) and were less likely to give up (OR = 0.50; 95% CI 0.30-0.82) compared to nulliparous women.

**Table 5:** Proportion and Odds Ratio (OR) of the RAS utilization* by women who had an intrauterine device inserted after attending the reproductive planning clinic to access the contraceptive method at the referral service according to parity (n=378). Belo Horizonte, MG, Brazil, 2024

RAS Utilization Variables*	Parity		valor-p^‡^	Parity	
	Nulliparous	Primiparous/Multiparous		Nulliparous	Primiparous/ Multiparous
	n (%)^†^	n (%)^†^		ORna^§^ (CI95%)^||^	
Knows reference UBS^¶^ (n**=378)			0,001^††^		
Yes	130 (81.8)	203 (92.7)		Ref.^‡‡^	2.83 (1.48-5.42)^§§^
Registration at the UBS^¶^ (n**=334)			0.047^††^		
Yes	120 (92.3)	198 (97.1)		Ref.^‡‡^	2.75 (0.97-7.76)
Attended UBS^¶^ in the last year (n**=310)			0.052		
Yes	86 (72.3)	156 (81.7)		Ref.^‡‡^	1.71 (0.99-2.95)
Discussed RP^||||^ with professional (n**=377)			0.357		
Yes	54 (34.2)	85 (38.8)			
Consultation with a professional to choose an IUD^¶¶^ (n**=378)			0.947		
Yes	59 (37.1)	82 (37.4)			
Professional consulted (n**=113)			0.680		
Physician/doctor	21 (56.8)	40 (52.6)		Ref.^‡‡^	1.18 (0.54-2.60)
Nurse	16 (43.2)	36 (47.4)		
Number of professionals consulted (n**=378)			0.145		
None	88 (55.4)	106 (48.4)			0.86 (0.55-1.35)
One	56 (35.2)	78 (35.6)		Ref.^‡‡^	
Two or more	15 (9.4)	35 (16.0)			1.68 (0.84-3.36)
Knows the insertion in the UBS^¶^ (n**=334)			<0.0001^††^		
Yes	14 (10.8)	56 (27.5)		Ref.^‡‡^	3.14 (1.66-5.91)^§§^
Attempt to join the UBS^¶^ (n**=334)			0.003^††^		
Yes	26 (20.0)	72 (35.3)		Ref.^‡‡^	2.18 (1.3-3.65)^§§^
Source of information on IUDs^¶¶^ (n**=378)			0.002^††^		
Physician/doctor	9 (5.7)	21 (9.6)		Ref.^‡‡^	2.31 (0.67-7.94)
Nurse	5 (3.1)	27 (12.3)		Ref.^‡‡^	0.51 (0.22-1.13)
Others***	145 (91.2)	171 (78.1)			
Waiting time for IUD insertion (in months) (n**=378)			0.003^††^		
<1	14 (8.8)	33 (15.1)		Ref.^‡‡^	
1-3	106 (66.7)	105 (48.0)			0.42 (0.21-0.83)^§§^
4-6	24 (15.1)	43 (19.6)			0.76 (0.34-1.69)
7 or more	15 (9.4)	38 (17.3)			1.07 (0.45-2.55)
Number of IUD insertion attempts^¶¶^ (n**=376)			0.042^††^		
None	77 (48.7)	84 (38.5)		Ref.^‡‡^	
One	61 (38.6)	87 (39.9)			1.31 (0.83-2.05)
Two or more	20 (12.7)	47 (21.6)			2.15 (1.17-3.96)^§§^
Thought about giving up (n**=378)			0.006^††^		
Yes	44 (27.7)	35 (16.0)		Ref.^‡‡^	0.50 (0.30-0.82)^§§^

*RAS = Health Care System; ^†^n (%) = Absolute and percentage frequency; ^‡^Pearson’s chi-square test; ^§^ORna = Unadjusted Odds Ratio; ^||^95%CI = 95% Confidence Interval; ^¶^UBS = Primary Health Care Units; **n = Total number of people who answered the question; ^††^Statistically significant p-value (p<0.05); ^‡‡^Ref. = Reference category; ^§§^Odds ratios with statistical significance, identified by the 95%CI which does not include the null value (OR = 1); ^||||^RP = Reproductive Planning; ^¶¶^IUD = Intrauterine Device; ***Friend/Parents/Concierge, secretary and teacher

## Discussion

The findings of this study show that although most women are aware of and use PHC services, when it comes to using the services specifically to access the IUD, there is a significant reduction in access to guidance on RP, on the possibility of inserting the IUD in this service, and women’s lack of knowledge about the possibility of inserting the IUD in the UBS. In addition, this reduction was even greater for women with less schooling and nulliparous women. Furthermore, there was a low proportion of referrals by professionals for IUD insertion at the referral clinic and a higher proportion of women waiting more than a month.

This result confirms the hypothesis of this study, as it identifies barriers to the use of RAS for accessing the IUD, with a care path marked by women’s navigation through services, a phenomenon already described in other dimensions of healthcare, such as childbirth^37^ ^)^ and multiple contexts of sexual and reproductive healthcare^25^ ^,^ ^30^. It also confirms the hypothesis that it is more challenging to achieve insertion in the RAS among women in situations of greater social vulnerability, among those with less education, and especially those with lower parity. However, no differences were observed in the use of RAS services based on race/skin color.

In relation to the barriers to using the RAS to access the IUD in the municipality, women reported difficulties not yet reported in previous studies, such as the lack of referral to the reference service, not knowing about the possibility of insertion in the UBS itself, and the waiting time of between 1-3 months for insertion. These findings demonstrate difficulties in access and the fragilities of HCN services in ensuring sexual and reproductive rights around contraception, additional barriers to what has been reported in previous studies^2^ ^,^ ^11^ ^,^ ^25^.

The difficulty in accessing the IUD in the Belo Horizonte system, evidenced in this study, could be a reality in other large Brazilian municipalities, since more than half of the municipalities reported not making the IUD available in PHC services^11^. In addition, evidence from studies in other locations, including Minas Gerais, points to the fact that the procedure is restricted and requires a referral from a medical professional^8^ ^-^ ^9^ ^,^ ^38^, often specialists, who are not present in all PHC services, in addition to the inadequate knowledge of health professionals about the IUD^38^ ^-^ ^39^. These factors constitute an institutional barrier that affects the realization of the right.

Such reality is aggravated by non-compliance with the legal deadlines for accessing the method, as evidenced in this study, in which most women waited more than 30 days, not complying with what is established in the Reproductive Planning Law^40^, increasing the risk of unintended pregnancy. This delay may be related to the availability of the method in the services, as a national study assessing the availability of supplies in PHC for RP in 2012, 2014, and 2018 identified lower availability of the IUD in these services in all years^11^. This could also explain the greater lack of knowledge reported by women about the availability of the IUD in the UBS found in this study.

Still about barriers, it is noteworthy that the rate of not inserting the IUD on the day of the appointment also requires attention, even though no significant differences were identified between the groups in terms of the use of the RAS and the clinical criteria. The inability to rule out pregnancy was the main reason for not inserting the IUD in this study and could be overcome with educational strategies, reproductive counseling, and appropriate care pathways^41^.

The analysis of predisposing and facilitating factors, as outlined in Andersen’s Behavioral Model of Health Service Utilization^36^, revealed social inequalities in the use of the RAS for the insertion of the method. A relationship was observed between a higher level of schooling and the use of health services, corroborating findings from international studies^21^ ^,^ ^28^. This relationship can be explained by the fact that higher levels of education contribute to transforming female reproductive patterns by strengthening decision-making capacity, increasing access to information, and facilitating social ascension^42^. A previous study also indicated that these women were more knowledgeable about the IUD^23^. Additionally, low levels of education may hinder the use of the IUD due to difficulties in understanding the instructions provided by professionals^43^.

In addition to schooling, the study highlights inequalities related to parity, as multiparous women were more aware of and used the UBS for IUD insertion. However, they also waited and sought insertion more actively than nulliparous women. The better situation of multiparous women could be explained by the greater ease and opportunities in accessing health services for the demands of the maternal-child continuum of care, which extends from prenatal care to child follow-up^3^. This finding corroborates the results of previous international studies^44^ ^-^ ^45^. In addition, similar results were found in Brazil^46^, encompassing all contraceptive methods. These findings demonstrate a greater appreciation of reproductive health, with mothers having greater access to services.

Thus, younger, single women, who constitute many nulliparous women, are excluded from accessing and using services, even though they have demands for contraception. As well as referring to social inequality, this scenario can compromise the full exercise of reproductive rights and constitutes contraceptive injustice^47^.

An advancement of this study is that it analyzed data from a referral service for IUD insertion staffed by obstetric nurses. It is known that the work of nurses is strategic in expanding access to IUDs in PHC, mainly because of their territorial insertion within the SUS. The national guideline^7^ recognizes and recommends the work of these professionals to strengthen reproductive and family planning, considering the needs of people at different stages of the life cycle. By assigning these professionals the competencies for IUD insertion and prescription, their role in ensuring access to the method is reinforced, contributing to a reduction in unplanned pregnancies, unsafe abortions, and maternal and infant morbidity and mortality^48^.

This strategy has been implemented in some regions of the country, such as São Paulo^49^_,_ Florianópolis^50^_,_ Mato Grosso do Sul^51^_,_ and several municipalities in the Northeast. As a result, PHC nurses have been trained and carry out IUD insertions in these municipalities^14^. The consistent work of nurses in inserting IUDs in these locations has made it possible to increase access to IUDs, reduce waiting time to obtain the method, promote the right to free and informed choice, and contribute to a more effective RAS. In other countries, such as Australia^15^, the United States^16^, and India^17^, the practice of nurses is established and systematized in accessing IUDs.

In addition, a study on the quality of care provided by obstetric nurses carried out in São Paulo showed that over 98% of women who had an IUD inserted by obstetricians and obstetric nurses reported that they had received RP guidance before insertion and that, of these, 98.6% considered it sufficient for them to feel safe using the IUD^49^. Data from the Health Information System for Primary Care (SISAB, acronym in Portuguese) indicate that nurses are primarily responsible for individual consultations related to the IUD, accounting for approximately 76%^8^. Thus, the role of nurses in IUD insertion expands women’s opportunities for both knowledge of LARC methods and effective access to their use^7^.

However, in Belo Horizonte’s PHC, nurses are not included in the local care protocols for inserting and prescribing the IUD during nursing consultations, despite recommendations in ministerial regulations^7^ and Federal Nursing Council Resolution No. 690/2022^14^. This fact could contribute to the results of this study, highlighting barriers in the care path of women accessing the method in RAS health services, reinforcing the existence of programmatic and executive problems in the supply of the technique. It should be emphasized that consistent action by nurses in the insertion of IUDs would make it possible to increase access to long-acting reversible contraception, reduce waiting times, and provide a viable alternative for filling care gaps.

Despite advances in national and international public policies on women’s health^6^ and the expansion of access to contraceptive methods, including the IUD, within the SUS, challenges persist, such as the lack of information about the available contraceptive methods and the capacity of services to offer them. Overcoming these challenges is fundamental to guaranteeing women’s right to health and the full exercise of their reproductive rights in Brazil.

This study has limitations, such as the exclusive evaluation of women seen at a referral service for IUD insertion, which could be considered a selection bias, since those who attend appointments may be more motivated to use CM and the IUD. Thus, the results may not accurately reflect the experiences of women who have not sought these services for various reasons, such as a lack of knowledge, financial, geographical, or cultural difficulties. Therefore, the coordination of the RAS may be even worse than identified in the study, representing a conservative bias, as women’s difficulties in using the RAS to access the method may have been underestimated. Therefore, the generalization of the results should be handled with caution.

On the other hand, this methodology enabled the retrieval of information about their previous journey to the referral service for IUD insertion, including any previous barriers encountered in RAS. Interviewing them more comprehensively across the entire system of a large municipality could be costly and prone to various failures, as described in previous studies^52^. Another limitation was the data’s inability to refer to the use of health services in the RAS, which is part of access in the broadest sense, but is not a measure of access per se. However, the study advances by investigating the use of services to achieve the method in the Brazilian capital with a representative sample.

The findings of this study have implications for professional practice and health management, including the need to strengthen the articulation between national macro-policies and local actions, specifically in relation to the RAS agreement and reference services, and to create strategies for health services to overcome the maternal-infant logic that guides contraceptive provision conditional on maternity, guaranteeing effective access for all women, regardless of their gestational history. In addition, it is essential to step up efforts to improve women’s knowledge of the availability and accessibility of Sexual and Reproductive Health services in the RAS, reviewing municipal RP protocols to include nurses in the prescription, insertion, review, and maintenance of IUDs. This would expand service provision for primary healthcare users, ensuring women’s autonomy in making informed choices and reducing inequalities.

## Conclusion

The findings of this study point to new barriers to IUD access. They also point to social inequalities in the use of RAS services in relation to schooling and parity. This evidence reinforces the need for public policies that prioritize equity in access to contraception and recognize the strategic role of nurses in ensuring sexual and reproductive rights.

## Data Availability

The dataset of this article is available on the RLAE page in the SciELO Data repository, at the link https://www.enf.ufmg.br/pos/tese.php
