# Supplementary material for: Inequalities in the health care system utilization for intrauterine device insertion
Source: Rev Lat Am Enfermagem. 2026 Mar 30;34:e4804. doi: 10.1590/1518-8345.7951.4804 (PMC13035206; doi:10.1590/1518-8345.7951.4804)
Supplement: Translation

# Translation: Desigualdades na utilização da rede de atenção à saúde para inserção do dispositivo intrauterino^2^

**DOI:** 10.1590/1518-8345.7951.4805

**Authors:** Síntia Nascimento dos Reis, Mery Natali Silva Abreu, Fernanda Penido Matozinhos, Larissa Soares Santos, Ana Paula Lage Guimarães Vallerini, Mariana Santos Felisbino-Mendes

**Affiliations:** 1 Universidade Federal de Minas Gerais, Escola de Enfermagem, Departamento de Enfermagem Materno-Infantil e Saúde Pública, Belo Horizonte, MG, Brasil.; 2 Universidade Federal de Minas Gerais, Escola de Enfermagem, Departamento de Gestão em Saúde, Belo Horizonte, MG, Brasil.; 3 Bolsista do Conselho Nacional de Desenvolvimento Científico e Tecnológico (CNPq), Brasil.; 4 Hospital Sofia Feldman, Belo Horizonte, MG, Brasil.

**Keywords:** Contracepção Reversível de Longo Prazo, Dispositivo Intrauterino, Planejamento Familiar, Desigualdades de Saúde, Enfermagem Obstétrica, Atenção à Saúde.

## Abstract

**Objetivo:**

investigar a utilização da Rede de Atenção à Saúde por mulheres em idade reprodutiva para inserção do dispositivo intrauterino e analisar possíveis desigualdades conforme características sociodemográficas.

**Método:**

estudo transversal, aninhado à coorte prospectiva, com amostra aleatória de 515 mulheres ≥18 anos, conduzido em serviço de referência para inserção do dispositivo intrauterino, por enfermeiras obstétricas. Aplicou-se questionário estruturado face a face para obter informações relativas à utilização da rede para inserção do dispositivo intrauterino. A análise foi conduzida com base no Modelo Comportamental de Utilização de Serviços de Saúde. Utilizou-se regressão logística e multinomial para estimar *Odds Ratio* de utilização dos serviços da rede segundo fatores predisponentes raça/cor e paridade, e fator facilitador, escolaridade.

**Resultados:**

a maioria das mulheres conhece e utiliza os serviços de saúde da rede, principalmente as com baixa escolaridade e multíparas. Porém, a utilização desses para acessar o dispositivo intrauterino é menor, com baixo acesso à orientação sobre planejamento reprodutivo e sobre a inserção do dispositivo intrauterino nos serviços, ainda menor entre aquelas com menor escolaridade e nulíparas.

**Conclusão:**

a menor chance de utilização da rede para acessar o dispositivo intrauterino entre mulheres com menor escolaridade e nulíparas revela desigualdades sociais no acesso à contracepção.

## Destaques

**(1)** Desconhecimento sobre inserção de DIU na UBS pelas mulheres cadastradas. **(2)** Fragilidades na RAS dificultam a utilização equitativa do DIU. **(3)** Utilização do DIU reflete desigualdades relacionadas à condição de paridade. **(4)** Peregrinação na Rede de Atenção à Saúde para utilização do método DIU. **(5)** Um total de 25% das mulheres que acessam o serviço de inserção não concluem o procedimento.

## Introdução

O uso de contraceptivos no Brasil tem se concentrado em pílulas, laqueadura tubária e camisinha, nessa ordem^1^ ^-^ ^2^. Esse ranking tem sido descrito como obsoleto^2^ ^-^ ^3^, por se resumir a métodos de curta duração ou cirúrgicos^2^ ^-^ ^3^. Trata-se de métodos que exigem disciplina constante para o uso correto e consistente^4^, com maiores taxas de falha, ou que implicam riscos decorrentes de cirurgia, além do alto custo.

O uso dos *Long-Acting Reversible Contraception* (LARCs), como o Dispositivo Intrauterino (DIU), apresentava em 2020 uma taxa global de uso em torno de 17%^5^. No entanto, no Brasil, em 2013, a prevalência do uso do DIU por mulheres em idade reprodutiva foi 1,8%^1^ e, em 2019, aumentou para 4,4 %^2^. Assim, o uso do método permanece incipiente no país em relação à taxa global^6^.

No âmbito do Sistema Único de Saúde (SUS), a estruturação da Rede de Atenção à Saúde (RAS) tem como porta de entrada os serviços de Atenção Primária à Saúde (APS), nos quais a inserção do DIU está prevista^7^. No entanto, estudos apontam barreiras ao acesso e uso do método^8^ ^-^ ^11^. As mais frequentemente reportadas são a inserção restrita ao médico^8^ ^-^ ^10^ e a menor disponibilidade do DIU nestes serviços em relação a outros métodos^11^. 

Iniciativas de ampliação do acesso ao DIU de cobre no âmbito da APS foram previamente descritas, como a inclusão da atuação de enfermeiros^12^ ^-^ ^13^, conforme previsto em normativas federais^14^. Esta estratégia mostrou melhoria no acesso ao método, como a capacitação destes profissionais em alguns contextos brasileiros^12^ ^-^ ^13^ e em outros países como Austrália^15^, Estados Unidos^16^ e Índia^17^. No entanto, ainda se configura como uma iniciativa pouco sistematizada no território nacional. 

Belo Horizonte está entre as nove capitais brasileiras que disponibilizam todos os métodos contraceptivos previstos pelo Ministério da Saúde^18^. No município, a inserção do DIU é prevista na Unidade Básica de Saúde (UBS) por médico especialista, que não está presente nas 152 unidades existentes^19^, o que se constitui em barreira de acesso ao método. Por outro lado, o município conta com ambulatório de referência na RAS, no qual as inserções são realizadas por enfermeiras obstétricas capacitadas, com registro de 6.418 inserções de DIU em 2022 e lista de espera de até cinco meses para a sua efetivação^20^. Acessar as mulheres atendidas neste ambulatório poderia contribuir para a identificação do percurso de utilização dos serviços de saúde da RAS, além de possíveis barreiras enfrentadas por mulheres até alcançar a inserção do DIU.

O estudo do processo de utilização dos serviços de saúde pode se apoiar em modelos teóricos. O Modelo Comportamental de Utilização de Serviços de Saúde de Andersen^21^ é o mais utilizado na literatura^22^. Nele, o acesso está intimamente relacionado com a utilização dos serviços de saúde, que é compreendida como a entrada nos serviços e todo contato direto ou indireto com o serviço, caracterizada pela garantia do atendimento^23^ ^-^ ^24^. Além disso, prevê que a utilização dos serviços de saúde é afetada por fatores de diferentes domínios, como os predisponentes, facilitadores e os de necessidade^21^ ^,^ ^25^. Os predisponentes se referem às características sociodemográficas e culturais que tornam os indivíduos mais ou menos propensos a utilizar os serviços de saúde^21^ ^-^ ^22^ ^,^ ^25^, os facilitadores, às condições que tornam possível ou facilitam o acesso aos serviços de saúde^21^ ^-^ ^22^ ^,^ ^25^, e os de necessidade, ao estado de saúde percebido e avaliado que motiva o uso dos serviços em saúde^21^ ^-^ ^22^ ^,^ ^25^. 

Estudos de contracepção que abordam seus determinantes frequentemente reportam que o acesso a este direito sexual e reprodutivo ainda é marcado por desigualdades tanto em países desenvolvidos^26^ quanto em países subdesenvolvidos^27^ ^-^ ^28^, com maior uso de contracepção moderna entre mulheres mais ricas e com ensino superior^28^. No Brasil, a prevalência do uso de contraceptivos foi menor entre mulheres pretas e pardas, bem como entre aquelas residentes nas regiões Norte e Nordeste^1^. Outro estudo mostrou perfil sociodemográfico diferente entre as usuárias de diferentes tipos de métodos, no qual os LARCs foram mais acessados por mulheres com melhores condições socioeconômicas^2^, corroborando achados em outros países^29^. No entanto, estes estudos não tratam do percurso assistencial de utilização da RAS para o acesso aos métodos.

Observa-se uma escassez de estudos sobre o percurso assistencial que as usuárias do SUS fazem na RAS para acessar o DIU, bem como as desigualdades sociais no uso da RAS, previamente identificadas em outros contextos de cuidado^25^ ^,^ ^30^. A maioria dos estudos se restringe à investigação do uso dos métodos contraceptivos^1^ ^-^ ^2^ ^,^ ^31^. Esta lacuna científica dificulta a identificação das barreiras para a utilização do DIU na atualidade, contribuindo para a invisibilidade do problema e para a injustiça contraceptiva, uma vez que existe um alto interesse em utilizar o método pelas brasileiras^32^. Essas barreiras podem ser decorrentes de problemas programáticos e executivos na oferta do método, o que pode refletir a realidade de outros contextos nacionais e internacionais. 

Assim, a primeira hipótese deste estudo é que existem dificuldades das mulheres na utilização da RAS para acessar o DIU. E a segunda é que há maior dificuldade de mulheres com maior vulnerabilidade social para alcançar a inserção do DIU, tais como menor escolaridade, maior paridade e cor de pele preta/parda. Portanto, o objetivo deste estudo foi investigar a utilização da Rede de Atenção à Saúde por mulheres em idade reprodutiva para inserção do DIU e analisar possíveis desigualdades conforme características sociodemográficas.

## Método

### Tipo de estudo

Trata-se de estudo clínico epidemiológico do tipo transversal, aninhado a uma coorte prospectiva, conduzido em serviço de referência para inserção do DIU por enfermeiras obstétricas no município de Belo Horizonte, Minas Gerais.

### População, amostra, critérios de inclusão e exclusão

Para a coorte prospectiva, foi calculada uma amostra aleatória simples representativa para o município a partir do número de consultas de inserção do DIU realizadas pelo referido serviço no ano de 2022, estratificada por tipo de DIU (cobre e hormonal) e o momento de inserção (pós-parto e de intervalo). Utilizaram-se os parâmetros de descontinuidade como desfecho principal e a baixa escolaridade como exposição. O cálculo foi realizado para nível de significância de 5%, poder de 80%, taxa de descontinuidade de 46,2% no grupo de alta escolaridade (não exposto) e *Odds Ratio* de 1,71^33^, totalizando 444 mulheres com a inserção do DIU. A esse valor, houve um acréscimo de 10%, considerando possíveis perdas de seguimento, resultando em um total de 489 mulheres com inserção do DIU após a consulta. Utilizou-se o programa *OpenEpi* versão 3.01 para o cálculo amostral do estudo.

Foram elegíveis mulheres com idade igual ou superior a 18 anos, residentes no município de Belo Horizonte, nulíparas e multíparas, que atendiam aos critérios de elegibilidade para o uso do método contraceptivo ofertado^34^.

Foram excluídas as mulheres participantes que já utilizavam o DIU, com suspeita ou confirmação de gestação, ou comprometimento cognitivo, e as que realizaram a inserção do DIU no pós-parto (n = 30), tendo em vista o acesso diferenciado a essa modalidade, especialmente pela confirmação prévia da ausência de gestação e pela possibilidade de inserção durante a internação hospitalar. Também foram aplicados critérios específicos conforme o tipo de DIU. Para o DIU de cobre, excluíram-se mulheres com alergia ao cobre, câncer cervical ou endometrial, anomalias uterinas, estenose cervical, imunossupressão, uso de anticoagulantes ou distúrbios de coagulação^34^. No caso do DIU hormonal, foram excluídas aquelas cujo uso estava indicado para tratamento de comorbidades, como sangramento uterino anormal, endometriose, adenomiose, leiomiomatose ou hiperplasia endometrial^35^. 

Desta forma, do total de 545 mulheres que responderam ao questionário da linha de base, após aplicar os critérios de exclusão, foram estudadas 515 mulheres, independentemente da efetivação da inserção do método contraceptivo. 

### Variáveis de interesse 

As variáveis de desfechos neste estudo referem-se à utilização dos serviços da RAS para inserção do DIU, conforme proposto por Andersen^24^: conhecimento da UBS de referência; cadastro na UBS; atendimento na UBS nos últimos 12 meses; discutiu planejamento reprodutivo com profissional; consulta com profissional para a escolha do DIU; conhecimento se UBS insere DIU; pensou em desistir; tentativa de realizar a inserção do DIU na UBS (categorizadas em sim e não); número de tentativas de inserção do DIU nos serviços de saúde antes do atendimento; quantidade de profissionais que consultou para inserção (nenhum, um, dois ou mais); qual profissional consultou para inserção do DIU (enfermeiro, médico, outros); tempo aguardado para realizar a inserção do DIU (<1, 1-3, 4-6, 7 meses ou mais); e quem informou sobre a inserção do DIU no hospital (enfermeiro, médico, admissão maternidade, amigo/a parentes, internet; referência do serviço da UBS, outros). 

A seleção das variáveis de exposição também foi orientada pelo Modelo Comportamental de Utilização de Serviços de Saúde^24^. Portanto, as características sociodemográficas individuais foram consideradas fatores predisponentes^36^ e incluíram: raça/cor (branca, preta e parda) e paridade (nenhuma, uma a duas e três ou mais). Como fator facilitador, incluiu-se a escolaridade (0-12 e 13 ou mais anos de estudo). 

Outras covariáveis relativas às características sociodemográficas e reprodutivas foram utilizadas para descrever a população de estudo e suas necessidades em contracepção: faixa etária (18-24, 25-29, 30-34 e 35 anos ou mais); situação conjugal (solteira/viúva ou divorciada, casada/união estável); parceria sexual (sim, não); trabalho remunerado (sim, não); ocorrência de gravidez prévia (sim, não); gravidez foi planejada (sim e não); deseja ter mais filhos (sim, não); utiliza algum método atualmente (sim, não); idade na primeira gestação (menor que 18, 18-24 e 25 ou mais); nível de satisfação com o método atual (indiferente, satisfeita/muito satisfeita, insatisfeita/muito insatisfeita). Algumas variáveis não foram respondidas por todas as mulheres, e aquelas que se autodeclararam amarelas (n=10), indígenas (n=1) e não informaram sua raça/cor (n=7) foram excluídas das análises devido ao número muito baixo e não representativo das categorias. 

### Coleta de dados 

A coleta de dados da linha de base da coorte foi realizada entre setembro de 2023 e agosto de 2024, em uma instituição filantrópica com atendimentos prestados integralmente pelo SUS. O serviço é reconhecido como referência na atuação de enfermeiras obstétricas, especialmente na inserção do DIU no âmbito da atenção à saúde reprodutiva.

Para a coleta de dados, foi elaborado um questionário com base em questões de instrumentos consolidados, como a Pesquisa Nacional de Demografia e Saúde da Criança e da Mulher (PNDS) de 2006 e as edições da Pesquisa Nacional de Saúde (PNS), realizadas em 2013 e 2019. O questionário foi testado em um estudo-piloto com sete mulheres, o que possibilitou adequações para aprimorar a clareza e coerência, alcançando o instrumento para utilização na coleta de dados. Ao final, os questionários foram estruturados e organizados em 12 módulos temáticos: identificação; critérios de inclusão e exclusão; informações para contato no seguimento; processo decisório para uso do DIU; trajetória na Rede de Atenção à Saúde (RAS); conhecimento sobre o método; características sociodemográficas; história reprodutiva; saúde sexual e reprodutiva atual. 

As entrevistas foram conduzidas com uso do questionário, face a face, presencialmente, no dia agendado para a consulta de inserção do DIU, enquanto as usuárias aguardavam o atendimento. A seleção das participantes ocorreu por amostragem aleatória, a partir da agenda de marcações do dia, considerando a disponibilidade dos entrevistadores e os agendamentos programados. Após a realização do questionário e da consulta, foi realizada a confirmação da inserção do método. A coleta de dados ocorreu por meio do *software Research Electronic Data Capture* (REDCap). 

A equipe de coleta de dados foi previamente capacitada, com treinamentos teóricos e práticos, incluindo treinamento sobre o DIU. Foi composta por estudantes de graduação em Enfermagem, enfermeiras, enfermeiras obstétricas, mestrandas e doutorandas em Enfermagem. A capacitação envolveu a apresentação do projeto de pesquisa, a realização de oficina temática sobre o DIU, a exposição detalhada do manual de coleta, a explicação do questionário e treinamento prático com simulação de entrevistas, utilizando o *software REDCap* em dispositivos móveis (celulares e *tablets*). Durante a coleta de dados, foram promovidas reuniões mensais de monitoramento com a equipe para discutir dificuldades operacionais e implementar melhorias contínuas na qualidade dos dados obtidos. Esse processo teve como objetivo assegurar o correto preenchimento do questionário e a padronização da abordagem às participantes.

### Análise de dados

Primeiramente, descreveu-se a população do estudo segundo características sociodemográficas e reprodutivas, por meio de frequências absolutas (n) e relativas (%). Em seguida, a mesma análise descritiva foi conduzida para os grupos de mulheres que inseriram e não inseriram o DIU após a avaliação na consulta. Posteriormente, calculou-se a proporção das variáveis de utilização da RAS para o total de mulheres e para os grupos das que inseriram e das que não puderam inserir o DIU. 

No presente estudo, as características sociodemográficas raça/cor e paridade foram consideradas fatores predisponentes, e a escolaridade como fator facilitador ou capacitante para utilização da RAS para alcançar o DIU como método contraceptivo. Assim, analisou-se, entre as mulheres que alcançaram a inserção do DIU, se suas características sociodemográficas (cor da pele/raça autorreferida, paridade e escolaridade) estavam associadas às variáveis de utilização da RAS. Nesta etapa, utilizaram-se o teste qui-quadrado de Pearson e o teste exato de Fisher para avaliar diferenças estatísticas entre as proporções. Em seguida, seguiu-se com a estimativa da *Odds Ratio* não ajustada (ORna) somente para os fatores que, na análise descritiva, apresentaram um p < 0,2. Utilizou-se a regressão logística para as variáveis de utilização binárias e a logística multinomial para aquelas com mais de duas respostas. Todas as análises foram realizadas no *software* STATA versão 16.1.

### Aspectos éticos

O estudo foi conduzido de acordo com as diretrizes de ética nacionais e internacionais e aprovado pelo Comitê de Ética em Pesquisa da Universidade Federal de Minas Gerais (parecer n.º 6.074.135; CAAE: 68438523.60000.5149) e do Hospital Sofia Feldman (parecer n.º 6.091.977; CAAE: 68438523.6.3001.5132), respectivamente em 23 e 31 de maio de 2023. Todas as participantes assinaram o Termo de Consentimento Livre e Esclarecido antes das entrevistas, cujo parecer está anexado à presente submissão.

## Resultados

As características sociodemográficas e reprodutivas das mulheres estudadas, total e segundo a situação de inserção do DIU, encontram-se na Tabela 1. Observa-se que a maioria eram jovens, entre 18-24 anos (37,5%) e 25-29 anos (27,9%), se autodeclararam pretas e pardas (73,5%); eram solteiras, viúvas ou divorciadas (71,7%); e possuíam parceria sexual atual (77,1%). Além disso, a maioria das mulheres possuía até 12 anos de escolaridade e trabalhava.

Quanto à história reprodutiva, das 507 mulheres que responderam sobre gestação prévia, 59,4% afirmaram ter engravidado. Dessas, a idade na primeira gestação foi entre 18 e 25 anos para 59,2%, e a maioria não planejou a gestação (60,4%). De todas as entrevistadas, a maioria não desejava engravidar novamente e utilizava algum método contraceptivo no momento da consulta, principalmente preservativo e pílula. Também estavam satisfeitas/muito satisfeitas com o método atual (67,1%) e, ao serem questionadas sobre a troca do método atual pelo DIU, a maioria relatou buscar mais segurança (40,6%), seguida de praticidade (18,8%) e devido a efeitos colaterais (15,5%).

Conforme a Tabela 1, não houve diferença das características sociodemográficas e reprodutivas entre os grupos que alcançaram ou não a inserção na data da consulta. Ressalta-se que, a cada quatro mulheres que acessaram o serviço de referência, uma não teve sucesso na inserção do DIU, mesmo estando agendada para o procedimento (25,4%; n=130). Os principais motivos da não inserção foram a impossibilidade de descartar gestação (37,0%) e o desejo por outro tipo de DIU não disponível no serviço (24,0%).

**Tabela 1:** Proporção das características sociodemográficas e reprodutivas das mulheres atendidas no Ambulatório de planejamento reprodutivo segundo a situação de inserção do dispositivo intrauterino (n = 515). Belo Horizonte, MG, Brasil, 2024

Características sociodemográficas e reprodutivas	Total	Situação Inserção do DIU*				Valor- p^||^
		Não Inseriu		Inseriu		
	n (%)^†^	N^‡^	%^§^	N^‡^	%^§^	
**Idade (anos) (n^¶^=509)**						0,722
18-24	191 (37,5)	54	41,5	137	36,1	
25-29	142 (27,9)	35	27,0	107	28,2	
30-34	93 (18,3)	21	16,1	72	19,0	
35 ou mais	83 (16,3)	20	15,4	63	16,7	
**Cor autodeclarada (n^¶^=491)**						0,836
Branca	130 (26,4)	33	25,8	97	26,7	
Preta e parda	361 (73,6)	95	74,2	266	73,3	
**Situação conjugal (n^¶^=509)**						0,531
Solteira, viúva ou divorciada	365 (71,7)	96	73,9	269	71,0	
Casada ou união estável	144 (28,3)	34	26,1	110	29,0	
**Escolaridade (anos de estudo) (n^¶^=509)**						0,205
0 a 12	317 (62,3)	87	66,9	230	60,7	
13 ou mais	192 (37,7)	43	33,1	149	39,3	
**Trabalha atualmente (n^¶^=509)**						0,739
Sim	393 (77,2)	99	76,1	294	77,6	
**Gravidez prévia (n^¶^=507)**						0,261
Sim	301 (59,4)	82	63,6	219	57,9	
**Número de gravidezes (n^¶^=507)**						0,308
0	206 (40,6)	47	36,4	159	42,0	
1	168 (33,1)	51	39,5	117	30,9	
2	88 (17,4)	22	17,0	66	17,5	
3 ou mais	45 (8,9)	9	7,0	36	9,5	
**Gravidez planejada (n^¶^=303)**						0,358
Não	183 (60,4)	53	60,4	130	58,8	
**Idade na 1ª gestação (anos) (n^¶^=302)**						0,633
<18	66 (21,9)	15	18,3	51	23,2	
18-25	179 (59,3)	50	61,0	129	58,6	
26 ou mais	57 (18,8)	17	20,7	40	18,2	
**Não deseja ter mais filhos (n^¶^=508)**						0,440
Não	296 (58,3)	72	55,4	224	59,3	
Usa MC** atualmente (n^¶^=502)						0,080
Sim	356 (71,0)	83	64,8	273	73,0	
**Método atual (n^¶^=356)**						0,094
Preservativo	124 (34,9)	25	30,1	99	36,3	
Pílula combinada e ou minipílula	108 (30,3)	25	30,1	83	30,4	
Injetável	57 (16,0)	47	12,1	10	17,2	
DIU* cobre e hormonal	52 (14,6)	16	19,3	36	13,2	
Outros^††^	15 (4,2)	7	8,4	8	2,9	
Satisfação com o MC** atual (n^¶^=356)						0,003
Indiferente	49 (13,8)	10	12,1	39	14,3	
Satisfeita/muito satisfeita	239 (67,1)	49	59,0	190	69,6	
Insatisfeita/muito insatisfeita	68 (19,1)	24	28,9	44	16,1	
**Tem parceria sexual atual (n^¶^=507)**						0,542
Sim	391 (77,1)	102	79,1	289	76,4	

*DIU = Dispositivo Intrauterino; ^†^n(%) = Frequência absoluta e percentual; ^‡^N = Total da amostra; ^§^% = Percentual; ^||^Valor-p = Teste qui-quadrado de Pearson; ^¶^n = Número total de pessoas que responderam à pergunta; **MC = Método contraceptivo; ^††^Outros = Métodos naturais, pílula de emergência e método de amenorreia lactacional

Segundo a Tabela 2, em relação à utilização dos serviços de saúde da RAS até o dia da consulta no serviço de referência, observou-se que a maioria das mulheres conhecia a UBS de referência, tinha cadastro e buscou atendimento na UBS no último ano. Entretanto, apenas 34,6% participaram de uma consulta de Planejamento Reprodutivo (PR) antes de escolher o DIU; 74,4% tentaram realizar a inserção do dispositivo na UBS e somente 20,9% conheciam essa possibilidade. Daquelas que realizaram consulta com profissional de saúde para escolha do DIU, apenas 32,2% consultaram com o enfermeiro.

Ainda conforme a Tabela 2, o acesso ao ambulatório para inserção do DIU foi realizado por indicação de amigos, parentes e outros indivíduos (83,3%), com baixa proporção de encaminhamentos por profissionais dos serviços da RAS. Quanto ao tempo de espera, 57,8% das mulheres aguardaram entre 1-3 meses e 30,6%, 4 meses ou mais. Não foram observadas diferenças significativas na utilização da RAS em relação à inserção do DIU.

Em seguida, analisou-se a utilização da RAS por cor da pele/raça, escolaridade e paridade para as mulheres que alcançaram a inserção do DIU (n=363). Na Tabela 3, observa-se que não houve diferenças nas chances de utilização da RAS segundo a cor da pele/raça. 

Os resultados da Tabela 4 mostram a utilização da RAS segundo escolaridade, na qual observa-se que mulheres com maior escolaridade, em comparação às de menor escolaridade, apresentaram maior utilização dos serviços da UBS no último ano (OR = 3,21; IC95%: 1,84-5,60), porém tiveram menor chance de ser atendidas em consulta específica para escolha do DIU (OR = 0,61; IC95%: 0,40-0,93).

**Tabela 2:** Proporção da utilização da RAS* pelas mulheres atendidas no ambulatório de planejamento reprodutivo para acessar o dispositivo intrauterino no serviço de referência segundo a situação de inserção do dispositivo intrauterino (n = 515). Belo Horizonte, MG, Brasil, 2024

Variáveis de Utilização da RAS*	Total	Situação Inserção do DIU^†^				valor-p^¶^
		Não Inseriu		Inseriu		
	n (%)^‡^	N^§^	%^||^	N^§^	%^||^	
Conhece UBS** de referência (n^††^=509)						0,919
Sim	449 (88,2)	115	88,5	334	88,1	
Cadastro na UBS** (n^††^=450)						0,426
Sim	431 (95,8)	112	97,4	319	95,2	
Atendida na UBS** no último ano (n^††^=423)						0,924
Sim	331 (78,2)	88	78,6	243	78,1	
**Discutiu PR^‡‡^ com profissional (n^††^=508)**						0,060
Sim	175 (34,6)	36	27,7	139	36,7	
**Consulta com profissional para a escolha do DIU^†^ (n^††^=509)**						0,955
Sim	320 (62,9)	82	63,1	238	62,8	
**Profissional consultado (n^††^=246)**						0,612
Médico	167 (67,8)	40	63,5	127	69,4	
Enfermeiro	79 (32,2)	23	36,5	56	30,6
**Número de profissionais consultados (n^††^=506)**						0,216
Nenhum	260 (51,4)	66	51,2	194	51,5	
Um	187 (36,9)	53	41,1	134	35,5	
Dois ou mais	59 (11,7)	10	7,7	49	13,0	
Conhece a inserção na UBS** (n^††^=450)	0,995
Sim	94 (20,9)	24	20,9	70	21,0	
Tentativa de inserção na UBS** (n^††^=450)						0,910
Sim	335 (74,4)	98	21,7	237	52,7	
**Fonte de informação do DIU^†^ (n^††^=509)**						0,939
Médico	41 (8,1)	11	8,5	30	7,9	
Enfermeiro	44 (8,6)	12	9,3	32	8,4	
Outros^§§^	424 (83,3)	107	82,3	317	83,6	
**Tempo aguardado para inserção do DIU^†^ (em meses) (n^††^=509)**						0,040
<1	59 (11,6)	11	8,5	48	12,6	
1-3	294 (57,8)	83	63,9	211	55,7	
4-6	79 (15,5)	12	9,2	67	17,7	
7 ou mais	77 (15,1)	24	18,5	53	14,0	
**Número de tentativas de inserção do DIU^†^ (n^††^=507)**						0,888
Nenhuma	215 (42,4)	53	40,8	162	43,0	
Uma	202 (39,8)	54	41,5	148	39,3	
Duas ou mais	90 (17,8)	23	17,7	67	17,8	
**Pensou em desistir (n^††^=509)**						0,281
Sim	112 (22,0)	33	25,4	79	20,8	

*RAS = Rede de Atenção à Saúde; ^†^DIU = Dispositivo Intrauterino; ^‡^n(%) = Frequência absoluta e percentual; ^§^N = Total da amostra; ^||^% = Percentual; ^¶^Valor-p = Teste qui-quadrado de Pearson; **UBS = Unidade Básica de Saúde; ^††^n = Número total de pessoas que responderam à pergunta; ^‡‡^PR = Planejamento Reprodutivo; ^§§^Outros = Amigo/Parentes/Porteiro, secretária e professora

**Tabela 3:** Proporção e *Odds Ratio* (OR) de utilização da RAS* por mulheres que inseriram o dispositivo intrauterino após o atendimento no Ambulatório de planejamento reprodutivo para acessar o método contraceptivo no serviço de referência de acordo com raça/cor (n = 363). Belo Horizonte, MG, Brasil, 2024

Variáveis de Utilização da RAS*	Raça/Cor		valor-p^‡^	Raça/Cor	
	Branca	Preta/Parda		Branca	Preta/Parda
	n (%)^†^	n (%)^†^		ORna^§^ (IC95%)^||^	
Conhece UBS^¶^ de referência (n**=363)			0,357		
Sim	83 (85,6)	237 (89,1)			
Cadastro na UBS^¶^ (n**=321)			0,275			
Sim	77 (92,8)	228 (95,8)			
Atendida na UBS^¶^ no último ano (n**=298)			0,234		
Sim	56 (73,7)	178 (80,2)			
Discutiu PR^††^ com profissional (n**=363)			0,719		
Sim	37 (38,1)	96 (36,1)		Ref.^‡‡^	1,09 (0,68-1,76)
Consulta com profissional para a escolha do DIU^§§^ (n**=363)			0,820			
Sim	37 (38,1)	98 (36,8)			
Profissional consultado (n**=108)			0,683			
Médico	13 (52,0)	47 (56,6)			
Enfermeiro	12 (48,0)	36 (43,4)		Ref.^‡‡^	1,21 (0,49-2,95)
Número de profissionais consultados (n**=363)			0,623			
Nenhum	49 (50,5)	137 (51,9)			
Um	38 (39,2)	93 (35,0)		Ref.^‡‡^	1,14 (0,69-1,88)
Dois ou mais	10 (10,3)	36 (13,5)		Ref.^‡‡^	0,78 (0,36-1,68)
Conhece a inserção na UBS^¶^ (n**=321)			0,334			
Sim	14 (16,9)	52 (21,9)			
Tentativa de inserção na UBS^¶^ (n**=321)			0,485			
Sim	26 (31,3)	65 (27,3)		Ref.^‡‡^	0,82 (0,48-1,42)
Fonte de informação do DIU^§§^ (n**=363)			0,619			
Médico	21 (7,9)	6 (6,2)			
Enfermeiro	20 (7,5)	10 (10,3)		Ref.^‡‡^	1,75 (0,54-5,71)
Outros^||||^	225 (84,6)	81 (83,5)		Ref.^‡‡^	1,26 (0,49-3,23)
Tempo aguardado para inserção do DIU^§§^ (em meses) (n**=363)			0,232			
<1	13 (13,4)	33 (12,4)			
1-3	52 (53,6)	153 (57,5)			
4-6	22 (22,7)	39 (14,1)			
7 ou mais	10 (19,6)	41 (80,4)			
Número de tentativas de inserção do DIU^§§^ (n**=361)			0,972			
Nenhuma	42 (43,7)	114 (43,1)			
Uma	38 (39,6)	104 (39,2)			
Duas ou mais	16 (16,7)	47 (17,7)			
Pensou em desistir (n**=363)			0,124			
Sim	25 (25,8)	49 (18,4)		Ref.^‡‡^	1,54 (0,89-2,67)

*RAS = Rede de Atenção à Saúde; ^†^n (%) = Frequência absoluta e percentual; ^‡^Teste qui-quadrado de Pearson; ^§^ORna = *Odds Ratio* não ajustada; ^||^IC95% = Intervalo de 95% de Confiança; ^¶^UBS = Unidade Básica de Saúde; **n = Número total de pessoas que responderam à pergunta; ^††^PR = Planejamento Reprodutivo; ^‡‡^Ref. = Categoria de referência; ^§§^DIU = Dispositivo Intrauterino; ^||||^Amigo/Parentes/Porteiro, secretária e professora

**Tabela 4:** Proporção e *Odds Ratio* (OR) de utilização da RAS* por mulheres que inseriram o dispositivo intrauterino após o atendimento no Ambulatório de planejamento reprodutivo para acessar o método contraceptivo no serviço de referência de acordo com a escolaridade (n = 379). Belo Horizonte, MG, Brasil, 2024

Variáveis de Utilização da RAS*	Escolaridade (em anos)		valor-p^‡^	Escolaridade (em anos)	
	0 a 12	13 ou mais		0 a 12	13 ou mais
	n (%)^†^	n (%)^†^		ORna^§^ (IC95%)^||^	
Conhece UBS^¶^ de referência (n**=379)			0,282			
Sim	206 (89,6)	128 (86,0)			
Cadastro na UBS^¶^ (n**=335)			0,333			
Sim	198 (96,1)	121 (93,8)			
Atendida na UBS^¶^ no último ano (n**=311)			<0,0001^††^			
Sim	78 (65,6)	165 (85,9)		Ref.^‡‡^	3,21 (1,84-5,60)^§§^
Discutiu PR^||||^ com profissional (n**=378)			0,073			
Sim	63 (42,3)	76 (33,2)		Ref.^‡‡^	0,68 (0,44-1,04)
Consulta com profissional para a escolha do DIU^¶¶^ (n**=379)			0,021^††^			
Sim	66 (44,3)	75 (32,6)		Ref.^‡‡^	0,61 (0,40-0,93)^§§^
Profissional consultado (n**=113)			0,342			
Médico	24 (60,0)	37 (50,7)		Ref.^‡‡^	
Enfermeiro	16 (40,0)	36 (49,3)			1,46 (0,67-3,19)
Número de profissionais consultados (n**=379)			0,090			
Nenhum	66 (44,3)	128 (55,7)		Ref.^‡‡^	
Um	59 (39,6)	75 (32,6)			0,66 (0,42-1,03)
Dois ou mais	24 (16,1)	27 (11,7)			0,58 (0,31-1,08)
Conhece a inserção na UBS^¶^ (n**=335)			0,414			
Sim	24 (18,6)	46 (22,3)			
Tentativa de inserção na UBS^¶^ (n**=335)			0,096			
Sim	31 (24,0)	67 (32,52)		Ref.^‡‡^	1,52 (0,93-2,51)
Fonte de informação do DIU (n**=379)			0,783			
Médico	11 (7,4)	19 (8,3)		Ref.^‡‡^	
Enfermeiro	11 (7,4)	21 (9,1)			1,10 (0,39-3,13)
Outros***	127 (85,2)	190 (82,6)			0,87 (0,40-1,88)
Tempo aguardado para inserção do DIU^¶¶^ (em meses) (n**=379)			0,015^††^			
<1	24 (16,1)	24 (10,4)		Ref.^‡‡^	
1-3	74 (49,7)	137 (59,6)			1,85 (0,98-3,48)
4-6	35 (23,5)	32 (13,9)			0,91 (0,44-1,92)
7 ou mais	16 (10,7)	37 (16,1)			2,31 (1,02-5,22)^§§^
Número de tentativas de inserção do DIU^¶¶^ (n**=377)			0,043^††^			
Nenhuma	61 (41,2)	101 (44,1)		Ref.^‡‡^	
Uma	68 (45,9)	80 (34,9)			0,71 (0,45-1,12)
Duas ou mais	19 (12,8)	48 (21,0)			1,56 (0,82-2,83)
Pensou em desistir (n**=379)			0,988			
Sim	48 (28,9)	31 (20,8)			

*RAS = Rede de Atenção à Saúde; ^†^n (%) = Frequência absoluta e percentual; ^‡^Teste qui-quadrado de Pearson; ^§^ORna = *Odds Ratio* não ajustada; ^||^IC95% = Intervalo de 95% de Confiança; ^¶^UBS = Unidade Básica de Saúde; **n = Número total de pessoas que responderam à pergunta; ^††^Valor-p estatisticamente significativo (p<0,05); ^‡‡^Ref.= Categoria de referência; ^§§^ *Odds ratios* com significância estatística, identificadas pelo IC95% que não inclui o valor nulo (OR = 1); ^||||^PR = Planejamento Reprodutivo; ^¶¶^DIU = Dispositivo Intrauterino; ***Amigo/Parentes/Porteiro, secretária e professora

Em relação à análise da utilização da RAS segundo paridade, de acordo com a Tabela 5, as multíparas têm maior chance de conhecer a UBS de referência (OR=2,83; IC95%1,48-5,42) e de conhecimento sobre a inserção de DIU na UBS (OR=3,14; IC95% 1,66-5,91) se comparadas com as nulíparas. Porém, essas mulheres tiveram menor chance de esperar 1-3 meses para a inserção do DIU (OR=0,42; IC95% 0,21-0,83) em comparação às nulíparas. Além disso, tiveram maior chance de um número maior de tentativas de inserção (2 ou mais tentativas: OR= 2,15; IC95% 1,17-3,96) e apresentaram menor chance de desistir (OR= 0,50; IC95% 0,30-0,82), se comparadas às nulíparas.

**Tabela 5:** Proporção e *Odds Ratio* (OR) de utilização da RAS* por mulheres que inseriram o dispositivo intrauterino após o atendimento no Ambulatório de planejamento reprodutivo para acessar o método contraceptivo no serviço de referência de acordo com a paridade (n = 378). Belo Horizonte, MG, Brasil, 2024

Variáveis de Utilização da RAS*	Paridade		valor-p^‡^	Paridade	
	Nulípara	Primípara/Multípara		Nulípara	Primípara/Multípara
	n (%)^†^	n (%)^†^		ORna^§^ (IC95%)^||^	
Conhece UBS^¶^ de referência (n**=378)			0,001^††^			
Sim	130 (81,8)	203 (92,7)		Ref.^‡‡^	2,83 (1,48-5,42)^§§^
Cadastro na UBS^¶^ (n**=334)			0,047^††^			
Sim	120 (92,3)	198 (97,1)		Ref.^‡‡^	2,75 (0,97-7,76)
Atendida na UBS^¶^ no último ano (n**=310)			0,052			
Sim	86 (72,3)	156 (81,7)		Ref.^‡‡^	1,71 (0,99-2,95)
Discutiu PR^||||^ com profissional (n**=377)			0,357			
Sim	54 (34,2)	85 (38,8)			
Consulta com profissional para a escolha do DIU^¶¶^ (n**=378)			0,947			
Sim	59 (37,1)	82 (37,4)			
Profissional consultado (n**=113)			0,680			
Médico	21 (56,8)	40 (52,6)		Ref.^‡‡^	1,18 (0,54-2,60)
Enfermeiro	16 (43,2)	36 (47,4)			
Número de profissionais consultados (n**=378)			0,145			
Nenhum	88 (55,4)	106 (48,4)			0,86 (0,55-1,35)
Um	56 (35,2)	78 (35,6)		Ref.^‡‡^	
Dois ou mais	15 (9,4)	35 (16,0)			1,68 (0,84-3,36)
Conhece a inserção na UBS^¶^ (n**=334)			<0,0001^††^			
Sim	14 (10,8)	56 (27,5)		Ref.^‡‡^	3,14 (1,66-5,91)^§§^
Tentativa de inserção na UBS^¶^ (n**=334)			0,003^††^			
Sim	26 (20,0)	72 (35,3)		Ref.^‡‡^	2,18 (1,3-3,65)^§§^
Fonte de informação do DIU^¶¶^ (n**=378)			0,002^††^			
Médico	9 (5,7)	21 (9,6)		Ref.^‡‡^	2,31 (0,67-7,94)
Enfermeiro	5 (3,1)	27 (12,3)		Ref.^‡‡^	0,51 (0,22-1,13)
Outros***	145 (91,2)	171 (78,1)			
Tempo aguardado para inserção do DIU^¶¶^ (em meses) (n**=378)			0,003^††^			
<1	14 (8,8)	33 (15,1)		Ref.^‡‡^	
1-3	106 (66,7)	105 (48,0)			0,42 (0,21-0,83)^§§^
4-6	24 (15,1)	43 (19,6)			0,76 (0,34-1,69)
7 ou mais	15 (9,4)	38 (17,3)			1,07 (0,45-2,55)
Número de tentativas de inserção do DIU^¶¶^ (n**=376)			0,042^††^			
Nenhuma	77 (48,7)	84 (38,5)		Ref.^‡‡^	
Uma	61 (38,6)	87 (39,9)			1,31 (0,83-2,05)
Duas ou mais	20 (12,7)	47 (21,6)			2,15 (1,17-3,96)^§§^
Pensou em desistir (n**=378)			0,006^††^			
Sim	44 (27,7)	35 (16,0)		Ref.^‡‡^	0,50 (0,30-0,82)^§§^

*RAS = Rede de Atenção à Saúde; ^†^n (%) = Frequência absoluta e percentual; ^‡^Teste qui-quadrado de Pearson; ^§^ORna = *Odds Ratio* não ajustada; ^||^IC95% = Intervalo de 95% de Confiança; ^¶^UBS = Unidade Básica de Saúde; **n = Número total de pessoas que responderam à pergunta; ^††^Valor-p estatisticamente significativo (p<0,05); ^‡‡^Ref. = Categoria de referência; ^§§^ *Odds ratios* com significância estatística, identificadas pelo IC95% que não inclui o valor nulo (OR = 1); ^||||^PR = Planejamento Reprodutivo; ^¶¶^DIU = Dispositivo Intrauterino; ***Amigo/Parentes/Porteiro, secretária e professora

## Discussão

Os achados do presente estudo apontam que, apesar de a maioria das mulheres conhecerem e utilizarem os serviços da APS, quando se trata da utilização dos serviços especificamente para acessar o DIU, há uma redução importante do acesso à orientação sobre PR, sobre a possibilidade de inserção do DIU neste serviço, e o desconhecimento das mulheres quanto à possibilidade de inserção do DIU na UBS. Adicionalmente, esta redução foi ainda maior para as mulheres com menor escolaridade e nulíparas. Além disso, houve baixa proporção de encaminhamentos por profissionais para a inserção do DIU no ambulatório de referência e maior proporção de mulheres que aguardam mais de um mês.

Este resultado confirma a hipótese deste estudo, pois aponta barreiras na utilização da RAS para o acesso ao DIU, com uma trajetória assistencial marcada por peregrinação das mulheres pelos serviços, fenômeno já descrito em outras dimensões da atenção à saúde, como em relação ao parto^37^ e outros contextos de cuidados à saúde sexual e reprodutiva^25^ ^,^ ^30^. Também confirma a hipótese de maior dificuldade de alcançar a inserção do DIU na RAS entre mulheres em situação de maior vulnerabilidade social, entre aquelas com menor escolaridade e principalmente as com menor paridade. No entanto, não foram observadas desigualdades na utilização dos serviços da RAS segundo raça/cor da pele. 

Em relação às barreiras para utilização da RAS para acessar o DIU no município, as mulheres relataram dificuldades ainda não reportadas em estudos prévios, tais como a ausência de referenciamento para o serviço de referência, o desconhecimento da possibilidade de inserção na própria UBS e o tempo de espera entre 1-3 meses para a inserção. Esses achados demonstram dificuldade de acesso e fragilidade dos serviços da RAS em garantir os direitos sexuais e reprodutivos no âmbito da contracepção, barreiras adicionais ao que foi reportado em estudos prévios^2^ ^,^ ^11^ ^,^ ^25^.

A dificuldade de acessar o DIU na rede de Belo Horizonte, evidenciada no presente estudo, poderia ser realidade em outros municípios brasileiros de grande porte, uma vez que mais da metade dos municípios referiu não disponibilizar o DIU nos serviços da APS^11^. Além disso, evidências de estudos em outras localidades, incluindo Minas Gerais, apontam para o fato de que a realização do procedimento é restrita e prescinde indicação do profissional médico^8^ ^-^ ^9^ ^,^ ^38^, muitas vezes especialistas, que não estão presentes em todos os serviços da APS, além do conhecimento inadequado dos profissionais de saúde acerca do DIU^38^ ^-^ ^39^. Tais fatores constituem uma barreira institucional que impacta a efetivação do direito.

Essa realidade é agravada pelo descumprimento dos prazos legais para acesso ao método, como evidenciado neste estudo, em que a maioria das mulheres aguardou mais de 30 dias, descumprindo o que está estabelecido na Lei do Planejamento Reprodutivo^40^, e amplia o risco de gravidez não intencional. Essa demora pode estar relacionada à disponibilidade do método nos serviços, pois estudo nacional que avaliou a disponibilidade de insumos nas APS para o PR em 2012, 2014 e 2018 identificou, em todos os anos, menor disponibilidade do DIU nestes serviços^11^. Tal fato também poderia justificar o maior desconhecimento reportado pelas mulheres sobre a disponibilidade do DIU nas UBS encontrada no presente estudo. 

Ainda em relação às barreiras, destaca-se que a taxa de não inserção do DIU no dia da consulta também demanda atenção, ainda que não tenham sido identificadas diferenças significativas entre os grupos quanto ao uso da RAS e aos critérios clínicos. A impossibilidade de descartar a gestação foi o principal motivo para a não inserção do DIU no presente estudo e poderia ser superada com estratégias educativas, aconselhamento reprodutivo e fluxos assistenciais adequados^41^. 

A análise dos fatores predisponentes e facilitadores, conforme previsto no Modelo Comportamental de Utilização de Serviços de Saúde de Andersen^36^, evidenciou desigualdades sociais na utilização da RAS para a inserção do método. Foi observada uma relação entre a maior escolaridade e a utilização dos serviços de saúde, corroborando achados de estudos internacionais^21^ ^,^ ^28^. Esta relação poderia ser explicada pelo fato de que níveis mais elevados de escolaridade contribuem para transformar padrões reprodutivos femininos ao fortalecer a capacidade de decisão, ampliar o acesso a informações e favorecer a ascensão social^42^. Estudo prévio também apontou maior conhecimento sobre o DIU destas mulheres^23^. Adicionalmente, a baixa escolaridade poderia obstar o uso do DIU pela dificuldade em compreender as orientações dos profissionais^43^.

Além da escolaridade, o estudo aponta para as desigualdades relacionadas à paridade, pois as multíparas tiveram maior conhecimento e utilização da UBS para a inserção do DIU. No entanto, também esperaram e buscaram mais ativamente a inserção no serviço do que as nulíparas. A melhor situação das multíparas poderia ser explicada pela maior facilidade e oportunidades em acessar os serviços de saúde para demandas da linha de cuidado contínuo materno-infantil, que se estende do pré-natal até o acompanhamento infantil^3^. Tal achado corrobora resultados de estudos prévios no cenário internacional^44^ ^-^ ^45^. Ademais, resultados semelhantes foram encontrados no cenário nacional^46^, porém englobando todos os métodos contraceptivos. Estes achados demonstram uma maior valorização da reprodução, no qual as mulheres que são mães têm maior acesso aos serviços. 

Assim, as mulheres mais jovens, solteiras, que configuram a maior parte das nulíparas, ficam alijadas do acesso e utilização dos serviços, mesmo tendo demandas por contracepção. Este cenário pode, além de remeter à desigualdade social, comprometer o exercício pleno dos direitos reprodutivos e configura-se como injustiça contraceptiva^47^.

Um avanço do presente estudo é ter analisado dados de um serviço de referência para inserção do DIU com atuação de enfermeiros obstétricos. Sabe-se que a atuação do enfermeiro é estratégica para ampliar o acesso ao DIU na APS, especialmente por sua inserção territorial no âmbito do SUS. A diretriz nacional^7^ reconhece e recomenda a atuação destes profissionais para o fortalecimento do planejamento reprodutivo e familiar, considerando as necessidades das pessoas ao longo de diferentes fases do ciclo de vida. Ao atribuir a esses profissionais competências para inserção e prescrição do DIU, reforça-se seu papel na garantia do acesso ao método, contribuindo para a redução de gestações não planejadas, abortos inseguros e da morbimortalidade materno-infantil^48^. 

Essa estratégia foi implementada em algumas regiões do país, como São Paulo^49^, Florianópolis^50^, Mato Grosso do Sul^51^ e vários municípios da região Nordeste. Com isso, as enfermeiras da APS foram capacitadas e realizam a inserção do DIU nesses municípios^14^. A atuação consistente dos enfermeiros na inserção do DIU nessas localidades possibilitou a ampliação do acesso ao DIU, reduzindo o tempo de espera para obtenção do método, promovendo o direito à escolha livre e informada e contribuindo para uma maior resolutividade da RAS. Em outros países, como Austrália^15^, Estados Unidos^16^ ^)^ e Índia^17^ a atuação da prática do enfermeiro encontra-se estabelecida e sistematizada no acesso ao DIU.

Ademais, estudo sobre a qualidade da assistência das enfermeiras obstétricas realizado em São Paulo mostrou que mais de 98% das mulheres que inseriram o DIU com obstetrizes e enfermeiras obstetras relataram que receberam orientação de PR antes da inserção e que, destas, 98,6% consideraram suficiente para que se sentissem seguras com o uso do DIU^49^. Dados do Sistema de Informação em Saúde para a Atenção Básica (SISAB) apontam que os enfermeiros são responsáveis pela maioria das consultas individuais relacionadas ao DIU, representando cerca de 76%^8^. Assim, a atuação dos enfermeiros na inserção do DIU amplia as oportunidades das mulheres tanto para o conhecimento dos métodos LARC quanto para o efetivo acesso à sua utilização^7^.

No entanto, na APS de Belo Horizonte, os enfermeiros não estão inseridos nos protocolos assistenciais locais para a inserção e prescrição do DIU durante a consulta de enfermagem, mesmo com as recomendações nas normativas ministeriais^7^ e na Resolução do Conselho Federal de Enfermagem N.º 690/2022^14^. Tal fato poderia contribuir para os resultados deste estudo, evidenciando barreiras no percurso assistencial das mulheres nos serviços de saúde da RAS para acessar o método, reforçando a existência de problemas programáticos e executivos na oferta do método. Ressalta-se que a atuação consistente dos enfermeiros na inserção do DIU possibilitaria a ampliação do acesso à contracepção reversível de longa duração, a redução do tempo de espera, além de uma alternativa viável para preencher vazios assistenciais.

Apesar dos avanços no âmbito de políticas públicas nacionais e internacionais nas políticas de saúde da Mulher^6^ e da ampliação do acesso aos métodos contraceptivos, incluindo o DIU, no âmbito do SUS, persistem desafios como a falta de informação em relação aos MC disponíveis e à capacidade dos serviços de ofertá-los. A superação destes desafios é fundamental para garantir a efetivação do direito à saúde das mulheres e ao exercício pleno de seus direitos reprodutivos no Brasil.

Este estudo apresenta limitações, tais como a avaliação exclusiva de mulheres atendidas em um serviço de referência para a inserção do DIU, o que poderia ser considerado um viés de seleção, uma vez que aquelas que comparecem às consultas podem ter maior motivação em relação ao uso de MC e o DIU. Assim, os resultados podem não refletir as experiências de mulheres que não procuraram esses serviços por diversos motivos, como desconhecimento, dificuldades financeiras, geográficas e culturais. Portanto, é possível que a coordenação da RAS esteja ainda pior do que a que foi identificada no estudo, representando um viés conservador, pois a dificuldade das mulheres em utilizar a RAS para acessar o método pode ter sido subestimada. Assim, a generalização dos resultados deve ser feita com cautela. 

Por outro lado, esta metodologia permitiu recuperar informações sobre o percurso prévio dessas usuárias até a chegada ao serviço de referência para inserção do DIU, incluindo barreiras anteriores na RAS. Entrevistá-las de forma mais abrangente em toda a rede de um município de grande porte poderia ser oneroso e propenso a diversas falhas, conforme descrito em estudos prévios^52^. Outro limite foi a capacidade dos dados se referirem à utilização dos serviços de saúde da RAS, que faz parte do acesso em sentido mais amplo, mas não se configura uma medida de acesso propriamente dito. No entanto, o estudo avança ao investigar a utilização dos serviços para alcançar o método em uma capital brasileira com amostra representativa. 

Os achados do presente estudo apresentam implicações para a prática profissional e a gestão em saúde, destacando-se a necessidade de fortalecer a articulação entre as macropolíticas nacionais e as ações locais, mais especificamente em relação à pactuação da RAS e dos serviços de referência, e criar estratégias para os serviços de saúde superarem a lógica materno-infantilista que orienta a oferta contraceptiva condicionada à maternidade, garantindo acesso efetivo a todas as mulheres, independentemente do histórico gestacional. Adicionalmente, é fundamental intensificar os esforços para melhorar o conhecimento das mulheres sobre a disponibilidade e acessibilidade dos serviços de Saúde Sexual e Reprodutiva da RAS, revisão dos protocolos municipais de PR para que incluam o enfermeiro na prescrição, inserção, revisão e manutenção do DIU. Assim, haveria expansão da oferta dos serviços para as usuárias da APS, assegurando a autonomia feminina na escolha livre informada e na redução das desigualdades.

## Conclusão

Os achados do presente estudo apontam novas barreiras no acesso ao DIU. Além disso, apontam desigualdades sociais na utilização da RAS do serviço em relação à escolaridade e à paridade. Tais evidências reforçam a necessidade de políticas públicas que priorizem a equidade no acesso à contracepção e reconheçam o papel estratégico dos enfermeiros na garantia dos direitos sexuais e reprodutivos.

## Declaração de Disponibilidade de Dados:

O conjunto de dados deste artigo está disponível na página da RLAE no repositório SciELO Data, no link https://www.enf.ufmg.br/pos/tese.php

# Translation: Desigualdades en la utilización de la red de atención a la salud para la inserción del dispositivo intrauterino^3^

**DOI:** 10.1590/1518-8345.7951.4803

**Authors:** Síntia Nascimento dos Reis, Mery Natali Silva Abreu, Fernanda Penido Matozinhos, Larissa Soares Santos, Ana Paula Lage Guimarães Vallerini, Mariana Santos Felisbino-Mendes

**Affiliations:** 1 Universidade Federal de Minas Gerais, Escola de Enfermagem, Departamento de Enfermagem Materno-Infantil e Saúde Pública, Belo Horizonte, MG, Brasil.; 2 Universidade Federal de Minas Gerais, Escola de Enfermagem, Departamento de Gestão em Saúde, Belo Horizonte, MG, Brasil.; 3 Becaria del Conselho Nacional de Desenvolvimento Científico e Tecnológico (CNPq), Brasil.; 4 Hospital Sofia Feldman, Belo Horizonte, MG, Brasil.

**Keywords:** Anticoncepción Reversible de Larga Duración, Dispositivos Intrauterinos, Planificación Familiar, Inequidades en Salud, Enfermería Obstétrica, Atención a la Salud.

## Abstract

**Objetivo:**

investigar la utilización de la Red de Atención a la Salud por mujeres en edad reproductiva para la inserción de dispositivos intrauterinos y analizar posibles desigualdades según características sociodemográficas.

**Método:**

estudio transversal, anidado en una cohorte prospectiva, con una muestra aleatoria de 515 mujeres ≥18 años, realizado en un servicio de referencia para la inserción de dispositivos intrauterinos, por enfermeras obstétricas. Se aplicó un cuestionario estructurado mediante entrevista cara a cara para obtener información relacionada con la utilización de servicios de salud para la inserción de dispositivos intrauterinos. El análisis se realizó basándose en el Modelo Comportamental de Uso de los Servicios de Salud. Se utilizó regresión logística y multinomial para estimar la *Odds Ratio* de uso de los servicios de la red según factores predisponentes como raza/color y paridad, y el factor facilitador, nivel educativo.

**Resultados:**

la mayoría de las mujeres conocen y utilizan los servicios de salud de la red, especialmente aquellas con bajo nivel educativo y multíparas. Sin embargo, el uso de estos servicios para acceder al dispositivo intrauterino es menor, con un acceso limitado a la orientación sobre planificación reproductiva y sobre la inserción del dispositivo intrauterino en los servicios, más bajo aún entre las mujeres con menor nivel educativo y nulíparas.

**Conclusión:**

la menor probabilidad de utilización de la red para acceder al dispositivo intrauterino entre las mujeres con menor nivel educativo y nulíparas revela desigualdades sociales en el acceso a la anticoncepción.

## Destacados

**(1)** Desconocimiento por parte de las mujeres registradas sobre la inserción del DIU en las UBS. **(2)** Las fragilidades en la RAS dificultan la utilización equitativa del DIU. **(3)** La utilización del DIU refleja desigualdades relacionadas con la condición de paridad. **(4)** Peregrinación en la Red de Atención a la Salud para la utilización del método DIU. **(5)** Un total del 25% de las mujeres que acceden al servicio de inserción no completan el procedimiento.

## Introducción

El uso de anticonceptivos en Brasil se ha concentrado en las pastillas, la ligadura de trompas y los condones, en ese orden^1^ ^-^ ^2^. Esta clasificzación ha sido descrita como obsoleta^2^ ^-^ ^3^, ya que se limita a métodos de corta duración o quirúrgicos^2^ ^-^ ^3^. Se trata de métodos que requieren disciplina constante para su uso correcto y consistente^4^, con mayores tasas de fracaso, o que implican riesgos derivados de la cirugía, además de un alto costo.

El uso de métodos anticonceptivos reversibles de acción prolongada (LARCs), como el dispositivo intrauterino (DIU), presentaba en 2020 una tasa global de uso de alrededor del 17%^(5^). Sin embargo, en Brasil, en 2013, la prevalencia del uso del DIU por parte de mujeres en edad reproductiva fue del 1,8% (1) y, en 2019, aumentó al 4,4%^2^. Por lo tanto, el uso de este método sigue siendo incipiente en el país en comparación con la tasa global^6^.

En el marco del Sistema Único de Salud (SUS), la estructuración de la Red de Atención a la Salud (RAS) tiene como puerta de entrada los servicios de Atención Primaria a la Salud (APS), en los que está prevista la inserción del DIU^7^. Sin embargo, algunos estudios señalan barreras para el acceso y el uso de este método^8^ ^-^ ^11^. Las más frecuentes son la inserción restringida al médico^8^ ^-^ ^10^ y la menor disponibilidad del DIU en estos servicios en comparación con otros métodos^11^.

Se han descrito anteriormente iniciativas para ampliar el acceso al DIU de cobre en el ámbito de la APS, como la inclusión de la actuación de enfermeros^12^ ^-^ ^13^, tal y como se prevé en la normativa federal^14^. Esta estrategia ha demostrado mejorar el acceso al método, como la capacitación de estos profesionales en algunos contextos brasileños^12^ ^-^ ^13^ ^)^ y en otros países como Australia^15^, Estados Unidos^16^ e India^17^. Sin embargo, todavía se configura como una iniciativa poco sistematizada en el territorio nacional.

Belo Horizonte se encuentra entre las nueve capitales brasileñas que ofrecen todos los métodos anticonceptivos previstos por el Ministerio de Salud^18^. En el municipio, la inserción del DIU se realiza en la Unidad Básica de Salud (UBS) por un médico especialista, que no está presente en las 152 unidades existentes^19^, lo que constituye una barrera de acceso al método. Por otro lado, el municipio cuenta con un consultorio de referencia en la RAS, en el que las inserciones son realizadas por enfermeras obstétricas capacitadas, con un registro de 6418 inserciones de DIU en 2022 y una lista de espera de hasta cinco meses para su realización^20^. El acceso a las mujeres atendidas en este consultorio podría contribuir a identificar el recorrido de utilización de los servicios de salud de la RAS, además de las posibles barreras a las que se enfrentan las mujeres hasta lograr la inserción del DIU.

El estudio del proceso de utilización de los servicios de salud puede basarse en modelos teóricos. El Modelo Conductual de Utilización de Servicios de Salud de Andersen^21^ es el más utilizado en la literatura^22^. En él, el acceso está íntimamente relacionado con el uso de los servicios de salud, que se entiende como el ingreso a los servicios y todo contacto directo o indirecto con el servicio, caracterizado por la garantía de la atención^23^ ^-^ ^24^. Además, prevé que el uso de los servicios de salud se ve afectado por factores de diferentes ámbitos, como los predisponentes, los facilitadores y los de necesidad^21^ ^,^ ^25^. Los predisponentes se refieren a las características sociodemográficas y culturales que hacen que los individuos sean más o menos propensos a utilizar los servicios de salud^21^ ^-^ ^22^ ^,^ ^25^, los facilitadores, a las condiciones que hacen posible o facilitan el acceso a los servicios de salud^21^ ^-^ ^22^ ^,^ ^25^, y los de necesidad, al estado de salud percibido y evaluado que motiva el uso de los servicios de salud^21^ ^-^ ^22^ ^,^ ^25^.

Los estudios sobre anticoncepción que abordan sus determinantes suelen indicar que el acceso a este derecho sexual y reproductivo sigue estando marcado por desigualdades tanto en los países desarrollados^26^ como en los subdesarrollados^27^ ^-^ ^28^, con un mayor uso de métodos anticonceptivos modernos entre las mujeres más ricas y con educación superior^28^. En Brasil, la prevalencia del uso de anticonceptivos fue menor entre las mujeres negras y morenas, así como entre las residentes en las regiones Norte y Noreste^1^. Otro estudio mostró un perfil sociodemográfico diferente entre las usuarias de diferentes tipos de métodos, en el que los LARC fueron más utilizados por mujeres con mejores condiciones socioeconómicas^2^, lo que corrobora los hallazgos en otros países^29^. Sin embargo, estos estudios no abordan la trayectoria asistencial del uso de la RAS para acceder a los métodos.

Se observa una escasez de estudios sobre el recorrido asistencial que realizan las usuarias del SUS en la RAS para acceder al DIU, así como las desigualdades sociales en el uso de la RAS, previamente identificadas en otros contextos de atención^25^ ^,^ ^30^. La mayoría de los estudios se limitan a investigar el uso de métodos anticonceptivos^1^ ^-^ ^2^ ^,^ ^31^. Esta laguna científica dificulta la identificación de las barreras para el uso del DIU en la actualidad, lo que contribuye a la invisibilidad del problema y a la injusticia anticonceptiva, ya que existe un gran interés en utilizar este método por parte de las mujeres brasileñas^32^. Estas barreras pueden deberse a problemas programáticos y ejecutivos en la oferta del método, lo que puede reflejar la realidad de otros contextos nacionales e internacionales.

Así, la primera hipótesis de este estudio es que las mujeres tienen dificultades para utilizar la RAS para acceder al DIU. Y la segunda es que las mujeres con mayor vulnerabilidad social, como aquellas con menor nivel educativo, mayor paridad y piel negra/ parda, tienen más dificultades para acceder a la inserción del DIU. Por lo tanto, el objetivo de este estudio fue investigar el uso de la Red de Atención a la Salud por parte de las mujeres en edad reproductiva para la inserción del DIU y analizar las posibles desigualdades según las características sociodemográficas.

## Método

### Tipo de estudio

Se trata de un estudio clínico epidemiológico transversal, integrado en una cohorte prospectiva, realizado en un servicio de referencia para la inserción de DIU por parte de enfermeras obstétricas en el municipio de Belo Horizonte, Minas Gerais.

### Población, muestra, criterios de inclusión y exclusión

Para la cohorte prospectiva, se calculó una muestra aleatoria simple representativa para el municipio a partir del número de consultas de inserción de DIU realizadas por dicho servicio en el año 2022, estratificada por tipo de DIU (cobre y hormonal) y momento de inserción (posparto e intervalo). Se utilizaron los parámetros de discontinuidad como resultado principal y el bajo nivel educativo como exposición. El cálculo se realizó para un nivel de significación del 5 %, un poder del 80 %, una tasa de discontinuidad del 46,2 % en el grupo de alto nivel educativo (no expuesto) y una *odds ratio* de 1,71^33^, lo que dio un total de 444 mujeres con inserción de DIU. A este valor se le añadió un 10% adicional, considerando posibles pérdidas de seguimiento, lo que dio como resultado un total de 489 mujeres con inserción de DIU después de la consulta. Se utilizó el programa *OpenEpi* versión 3.01 para el cálculo muestral del estudio.

Mujeres elegibles con edad igual o superior a 18 años, residentes en la ciudad de Belo Horizonte, nulíparas y multíparas, que cumplieran con los criterios de elegibilidad para el uso del método anticonceptivo ofrecido^34^.

Se excluyeron las mujeres participantes que ya usaban el DIU, con sospecha o confirmación de embarazo, o deterioro cognitivo, y aquellas que se sometieron a inserción del DIU en el posparto (n = 30), en vista del acceso diferenciado a esta modalidad, especialmente debido a la confirmación previa de la ausencia de embarazo y la posibilidad de inserción durante la hospitalización. También se aplicaron criterios específicos según el tipo de DIU. Para el DIU de cobre, se excluyeron las mujeres con alergia al cobre, cáncer de cuello uterino o de endometrio, anomalías uterinas, estenosis cervical, inmunosupresión, uso de anticoagulantes o trastornos de la coagulación^34^. En el caso del DIU hormonal, se excluyeron aquellas cuyo uso estaba indicado para el tratamiento de comorbilidades, como sangrado uterino anormal, endometriosis, adenomiosis, leiomiomatosis o hiperplasia endometrial^35^.

Así, del total de 545 mujeres que respondieron al cuestionario basal, después de aplicar los criterios de exclusión, se estudiaron 515 mujeres, independientemente de la inserción efectiva del método anticonceptivo.

### Variables de interés

Las variables de resultado de este estudio se refieren al uso de los servicios de la RAS para la inserción del DIU, según lo propuesto por Andersen^24^: conocimiento de la UBS de referencia; registro en el UBS; atención en la UBS en los últimos 12 meses; discutió la planificación reproductiva con un profesional; consulta con un profesional para elegir el DIU; conocimiento de si UBS inserta DIU; Pensó en darse por vencido; intentar insertar el DIU en el UBS (categorizado como sí y no); número de intentos de inserción del DIU en los servicios de salud antes de la atención; número de profesionales consultados para inserción (ninguno, uno, dos o más); a qué profesional consultó para la inserción del DIU (enfermera, médico, otros); tiempo de espera para insertar el DIU (<1, 1-3, 4-6, 7 meses o más); y quién informó sobre la inserción del DIU en el hospital (enfermera, médico, ingreso por maternidad, amigo de familiares, internet; referencia del servicio UBS, otros).

La selección de las variables de exposición también fue guiada por el Modelo Conductual de Uso de Servicios de Salud^24^. Por lo tanto, las características sociodemográficas individuales se consideraron factores predisponentes^36^ e incluyeron: raza/color (blanco, negro y marrón) y paridad (ninguno, uno a dos y tres o más). Como factor facilitador, se incluyó la escolaridad (0-12 y 13 o más años de escolaridad).

Se utilizaron otras covariables relacionadas con las características sociodemográficas y reproductivas para describir la población de estudio y sus necesidades anticonceptivas: grupo de edad (18-24, 25-29, 30-34 y 35 años o más); estado civil (soltero/viudo o divorciado, casado/unión estable); pareja sexual (sí, no); trabajo remunerado (sí, no); ocurrencia de embarazo previo (sí, no); el embarazo fue planificado (sí y no); quiere tener más hijos (sí, no); usa algún método actualmente (sí, no); edad del primer embarazo (menos de 18, 18-24 y 25 o más); Nivel de satisfacción con el método actual (indiferente, satisfecho/muy satisfecho, insatisfecho/muy insatisfecho). Algunas variables no fueron respondidas por todas las mujeres, y las que se autodeclararon amarillas (n=10), indígenas (n=1) y no informaron su raza/color (n=7) fueron excluidas de los análisis debido al número muy bajo y no representativo de categorías.

### Recogida de datos 

La recolección de datos de referencia de cohorte se realizó entre septiembre de 2023 y agosto de 2024, en una institución filantrópica con atención prestada íntegramente por el SUS. El servicio es reconocido como una referencia en el trabajo de las enfermeras obstétricas, especialmente en la inserción del DIU en el ámbito de la atención a la salud reproductiva.

Para la recolección de datos, se desarrolló un cuestionario basado en preguntas de instrumentos consolidados, como la Encuesta Nacional de Demografía y Salud de Niños y Mujeres (PNDS) de 2006 y las ediciones de la Encuesta Nacional de Salud (PNS), realizadas en 2013 y 2019. El cuestionario se probó en un estudio piloto con siete mujeres, lo que permitió realizar ajustes para mejorar la claridad y la coherencia, llegando al instrumento para su uso en la recolección de datos. Al final, los cuestionarios se estructuraron y organizaron en 12 módulos temáticos: identificación; criterios de inclusión y exclusión; información de contacto en el seguimiento; proceso de toma de decisiones para la utilización del DIU; trayectoria en la RAS; conocimiento sobre el método; características sociodemográficas; historia reproductiva; salud sexual y reproductiva actual.

Las entrevistas se realizaron mediante el cuestionario, cara a cara, en persona, el día programado para la cita de inserción del DIU, mientras las usuarias esperaban el servicio. La selección de los participantes se realizó por muestreo aleatorio, con base en el horario de citas del día, considerando la disponibilidad de los entrevistadores y las citas programadas. Después del cuestionario y la consulta, se confirmó la inserción del método. La recolección de datos se realizó utilizando el *software Research Electronic Data Capture* (*REDCap*).

El equipo de recolección de datos fue capacitado previamente, con capacitación teórica y práctica, incluida la capacitación sobre el DIU. Estaba compuesto por estudiantes de pregrado en enfermería, enfermeras, enfermeras obstétricas, estudiantes de maestría y estudiantes de doctorado en enfermería. La capacitación incluyó la presentación del proyecto de investigación, la realización de un taller temático sobre el DIU, la presentación detallada del manual de colecta, la explicación del cuestionario y la capacitación práctica con simulación de entrevistas, utilizando el *software REDCap* en dispositivos móviles (celulares y tabletas). Durante la recolección de datos, se realizaron reuniones mensuales de monitoreo con el equipo para discutir las dificultades operativas e implementar mejoras continuas en la calidad de los datos obtenidos. Este proceso tuvo como objetivo garantizar la correcta cumplimentación del cuestionario y la estandarización del enfoque a los participantes.

### Análisis de datos

En primer lugar, se describió la población de estudio de acuerdo con las características sociodemográficas y reproductivas, utilizando frecuencias absolutas (n) y relativas (%). Luego, se realizó el mismo análisis descriptivo para los grupos de mujeres que insertaron y no insertaron el DIU después de la evaluación en la consulta. Posteriormente, se calculó la proporción de las variables de uso de la RAS para el número total de mujeres y para los grupos de las que insertaron y las que no pudieron insertar el DIU.

En el presente estudio, se consideraron factores predisponentes las características sociodemográficas raza/color y paridad, y la educación como factor facilitador o habilitador para el uso de la RAS para alcanzar el DIU como método anticonceptivo. Así, entre las mujeres que lograron la inserción del DIU, se analizó si sus características sociodemográficas (color de piel/raza autorreferida, paridad y escolaridad) estaban asociadas con las variables de uso de la RAS. En este paso, se utilizó la prueba de chi-cuadrado de Pearson y la prueba exacta de Fisher para evaluar las diferencias estadísticas entre las proporciones. Luego, se estimó la *Odds Ratio* no ajustada (ORna) solo para los factores que, en el análisis descriptivo, presentaron un p < 0,2. Se utilizó regresión logística para las variables binarias de uso y logística multinomial para aquellas con más de dos respuestas. Todos los análisis se realizaron con el software STATA, versión 16.1.

### Aspectos éticos

El estudio fue realizado de acuerdo con las directrices éticas nacionales e internacionales y aprobado por el Comité de Ética en Investigación de la Universidad Federal de Minas Gerais (parecer nº 6.074.135; CAAE: 68438523.60000.5149) y Hospital Sofia Feldman (dictamen nº 6.091.977; CAAE: 68438523.6.3001.5132), respectivamente el 23 y 31 de mayo de 2023. Todos los participantes firmaron el Término de Consentimiento Informado antes de las entrevistas, cuya opinión se adjunta a este envío.

## Resultados

Las características sociodemográficas y reproductivas de las mujeres estudiadas, en su conjunto y de acuerdo con la situación de inserción del DIU, se muestran en la Tabla 1. Se observa que la mayoría eran jóvenes, entre 18-24 años (37,5%) y 25-29 años (27,9%), autodeclarados negros y pardos (73,5%); eran solteros, viudos o divorciados (71,7%); y tenía pareja sexual actual (77,1%). Además, la mayoría de las mujeres tenían hasta 12 años de escolaridad y trabajaban.

En cuanto a los antecedentes reproductivos, de las 507 mujeres que respondieron sobre embarazo previo, 59,4% afirmaron haber quedado embarazadas. De estos, la edad del primer embarazo fue entre los 18 y los 25 años para el 59,2%, y la mayoría no planificó su embarazo (60,4%). De todas las entrevistadas, la mayoría no quería volver a quedar embarazada y utilizaba algún método anticonceptivo en el momento de la consulta, principalmente preservativos y píldoras. También estaban satisfechas/muy satisfechas con el método actual (67,1%) y, cuando se les preguntó sobre el cambio del método actual al DIU, la mayoría informó que buscaba más seguridad (40,6%), seguida de la practicidad (18,8%) y debido a los efectos secundarios (15,5%).

De acuerdo con la Tabla 1, no hubo diferencia en las características sociodemográficas y reproductivas entre los grupos que lograron o no la inclusión en la fecha de la consulta. Cabe destacar que, por cada cuatro mujeres que accedieron al servicio de referencia, una no logró insertar el DIU, a pesar de que estaba programada para el procedimiento (25,4%; n=130). Los principales motivos de no inserción fueron la imposibilidad de descartar el embarazo (37,0%) y el deseo de otro tipo de DIU no disponible en el servicio (24,0%).

**Tabla 1:** Proporción de características sociodemográficas y reproductivas de las mujeres atendidas en el Ambulatorio de Planificación Reproductiva según la situación de inserción del dispositivo intrauterino (n = 515). Belo Horizonte, MG, Brasil, 2024

Características sociodemográficas y reproductivas	Total	Situación Inserción del DIU*				Valor p^||^
		No Insertado		Insertado		
	n (%)^†^	N^‡^	%^§^	N^‡^	%^§^	
**Edad (años) (n^¶^=509)**						0,722
18-24	191 (37,5)	54	41,5	137	36,1	
25-29	142 (27,9)	35	27,0	107	28,2	
30-34	93 (18,3)	21	16,1	72	19,0	
35 o más	83 (16,3)	20	15,4	63	16,7	
**Color autodeclarado (n^¶^=491)**						0,836
Blanco	130 (26,4)	33	25,8	97	26,7	
Negro y marrón	361 (73,6)	95	74,2	266	73,3	
**Estado civil (n^¶^=509)**						0,531
Soltera, viuda o divorciada	365 (71,7)	96	73,9	269	71,0	
Casada o en unión estable	144 (28,3)	34	26,1	110	29,0	
**Educación (años de estudio) (n^¶^=509)**						0,205
0 a 12	317 (62,3)	87	66,9	230	60,7	
13 o más	192 (37,7)	43	33,1	149	39,3	
**Actualmente trabaja (n^¶^=509)**						0,739
Sí	393 (77,2)	99	76,1	294	77,6	
**Embarazo previo (n^¶^=507)**						0,261
Sí	301 (59,4)	82	63,6	219	57,9	
**Número de embarazos (n^¶^=507)**						0,308
0	206 (40,6)	47	36,4	159	42,0	
1	168 (33,1)	51	39,5	117	30,9	
2	88 (17,4)	22	17,0	66	17,5	
3 o más	45 (8,9)	9	7,0	36	9,5	
**Embarazo planificado (n^¶^=303)**						0,358
No	183 (60,4)	53	60,4	130	58,8	
**Edad en el primer embarazo (años) (n^¶^=302)**						0,633
<18	66 (21,9)	15	18,3	51	23,2	
18-25	179 (59,3)	50	61,0	129	58,6	
26 o más	57 (18,8)	17	20,7	40	18,2	
**No desea tener más hijos (n^¶^=508)**						0,440
No	296 (58,3)	72	55,4	224	59,3	
Actualmente usa MC** (n^¶^=502)						0,080
Sí	356 (71,0)	83	64,8	273	73,0	
**Método actual (n^¶^=356)**						0,094
Condón	124 (34,9)	25	30,1	99	36,3	
Píldora combinada y\o minipíldora	108 (30,3)	25	30,1	83	30,4	
Inyectable	57 (16,0)	47	12,1	10	17,2	
DIU* de cobre y hormonales	52 (14,6)	16	19,3	36	13,2	
Otros^††^	15 (4,2)	7	8,4	8	2,9	
Satisfacción con el MC** actual (n^¶^=356)						0,003
Indiferente	49 (13,8)	10	12,1	39	14,3	
Satisfecha/muy satisfecha	239 (67,1)	49	59,0	190	69,6	
Insatisfecha/muy insatisfecha	68 (19,1)	24	28,9	44	16,1	
**Tiene una pareja sexual actual (n^¶^=507)**						0,542
Sí	391 (77,1)	102	79,1	289	76,4	

*DIU = Dispositivo Intrauterino; ^†^n(%) = Frecuencia absoluta y porcentaje; ^‡^N = Muestra total; ^§^% = Porcentaje; ^||^Valor-p = Prueba de chi-cuadrado de Pearson; ^¶^n = Número total de personas que respondieron la pregunta; **MC = Método anticonceptivo; ^††^Otros = Métodos naturales, píldora de emergencia y método de amenorrea de la lactancia

De acuerdo con la Tabla 2, en relación con el uso de los servicios de salud de la RAS hasta el día de la consulta en el servicio de referencia, se observó que la mayoría de las mujeres conocían la UBS de referencia, tenían un registro y buscaron atención en la UBS en el último año. Sin embargo, solo el 34,6% participó en una consulta de Planificación Reproductiva (PR) antes de elegir el DIU; El 74,4% intentó insertar el dispositivo en el UBS y solo el 20,9% conocía esta posibilidad. De las que tuvieron una consulta con un profesional de la salud para elegir el DIU, solo 32,2% tuvieron una consulta con enfermera.

También de acuerdo con la Tabla 2, el acceso a la consulta externa para la inserción del DIU se realizó por derivación de amigos, familiares y otras personas (83,3%), con una baja proporción de derivaciones por parte de profesionales de los servicios de la RAS. En cuanto al tiempo de espera, el 57,8% de las mujeres esperó entre 1-3 meses y el 30,6%, 4 meses o más. No se observaron diferencias significativas en el uso de la RAS en relación con la inserción del DIU.

A continuación, se analizó el uso de la RAS por color de piel/raza, educación y paridad para las mujeres que lograron la inserción del DIU (n = 363). La Tabla 3 muestra que no hubo diferencias en las probabilidades de usar la RAS según el color de la piel/raza. 

Los resultados de la Tabla 4 muestran el uso de la RAS según la educación, en la que se observa que las mujeres con mayor educación, en comparación con las que tienen menor educación, tuvieron mayor uso de los servicios de la UBS en el último año (OR = 3,21; IC95%: 1,84-5,60), pero tuvieron menos probabilidades de ser atendidas en una consulta específica para elegir el DIU (OR = 0,61; IC95%: 0,40-0,93).

**Tabla 2:** Proporción de uso de la RAS* por mujeres atendidas en el ambulatorio de planificación reproductiva para acceder al dispositivo intrauterino en el servicio de referencia según la situación de inserción del dispositivo intrauterino (n = 515). Belo Horizonte, MG, Brasil, 2024

Variables de Utilización de la RAS*	Total	Situación Inserción del DIU^†^				Valor p^¶^
		No Insertado		Insertado		
	n (%)^‡^	N^§^	%^||^	N^§^	%^||^	
Conoce UBS** de referencia (n^††^=509)						0,919
Sí	449 (88,2)	115	88,5	334	88,1	
Inscripción en la UBS** (n^††^=450)						0,426
Sí	431 (95,8)	112	97,4	319	95,2	
Asistida en la UBS** en el último año (n^††^=423)						0,924
Sí	331 (78,2)	88	78,6	243	78,1	
**Discutió PR^‡‡^ con un profesional (n^††^=508)**						0,060
Sí	175 (34,6)	36	27,7	139	36,7	
**Consulta con un profesional para elegir el DIU^†^ (n^††^=509)**						0,955
Sí	320 (62,9)	82	63,1	238	62,8	
**Profesional consultado (n^††^=246)**						0,612
Médico	167 (67,8)	40	63,5	127	69,4	
Enfermero	79 (32,2)	23	36,5	56	30,6	
**Número de profesionales consultados (n^††^=506)**						0,216
Ninguno	260 (51,4)	66	51,2	194	51,5	
Uno	187 (36,9)	53	41,1	134	35,5	
Dos o más	59 (11,7)	10	7,7	49	13,0	
Conozca la inserción en la UBS** (n^††^=450)						0,995
Sí	94 (20,9)	24	20,9	70	21,0	
Intento de inserción en la UBS** (n^††^=450)						0,910
Sí	335 (74,4)	98	21,7	237	52,7	
**Fuente de información sobre el DIU^†^ (n^††^=509)**						0,939
Médico	41 (8,1)	11	8,5	30	7,9	
Enfermero	44 (8,6)	12	9,3	32	8,4	
Otros^§§^	424 (83,3)	107	82,3	317	83,6	
**Tiempo esperado para la inserción del DIU^†^ (en meses) (n^††^=509)**						0,040
<1	59 (11,6)	11	8,5	48	12,6	
1-3	294 (57,8)	83	63,9	211	55,7	
4-6	79 (15,5)	12	9,2	67	17,7	
7 o más	77 (15,1)	24	18,5	53	14,0	
**Número de intentos de inserción del DIU^†^ (n^††^=507)**						0,888
Ninguno	215 (42,4)	53	40,8	162	43,0	
Uno	202 (39,8)	54	41,5	148	39,3	
Dos o más	90 (17,8)	23	17,7	67	17,8	
**Pensó en rendirse (n^††^=509)**						0,281
Sí	112 (22,0)	33	25,4	79	20,8	

*RAS = Red de Atención a la Salud; ^†^DIU = Dispositivo intrauterino; ^‡^n(%) = Frecuencia absoluta y porcentual; ^§^N = Muestra total; ^||^% = Porcentaje; ^¶^valor p = Prueba de chi-cuadrado de Pearson; **UBS = Unidad Básica de Salud; ^††^n = Número total de personas que respondieron la pregunta; ^‡‡^PR = Planificación reproductiva; ^§§^Otros = Amigo/Parientes/Portero, secretaria y profesora

**Tabla 3:** Proporción y *Odds Ratio* (OR) de uso de la RAS* por mujeres que insertaron el dispositivo intrauterino después de ser tratadas en el Ambulatorio de Planificación Reproductiva para acceder al método anticonceptivo en el servicio de referencia según raza/color (n = 363). Belo Horizonte, MG, Brasil, 2024

Variables de Utilización de la RAS*	Raza/Color		Valor p^‡^	Raza/Color	
	Blanco	Negro/Marrón		Blanco	Negro/Marrón
	n (%)^†^	n (%)^†^		ORna^§^ (IC95%)^||^	
Conoce UBS^¶^ de referencia (n**=363)			0,357		
Sim	83 (85,6)	237 (89,1)			
Inscripción en la UBS^¶^ (n**=321)			0,275		
Sim	77 (92,8)	228 (95,8)			
Asistida en la UBS^¶^ en el último año (n**=298)			0,234		
Sim	56 (73,7)	178 (80,2)			
Discutió PR^††^ con un profesional (n**=363)			0,719		
Sí	37 (38,1)	96 (36,1)		Ref.^‡‡^	1,09 (0,68-1,76)
Consulta con un profesional para elegir el DIU^§§^ (n**=363)			0,820		
Sí	37 (38,1)	98 (36,8)			
Profesional consultado (n**=108)			0,683		
Médico	13 (52,0)	47 (56,6)			
Enfermero	12 (48,0)	36 (43,4)		Ref.^‡‡^	1,21 (0,49-2,95)
Número de profesionales consultados (n**=363)			0,623		
Ninguno	49 (50,5)	137 (51,9)			
Uno	38 (39,2)	93 (35,0)		Ref.^‡‡^	1,14 (0,69-1,88)
Dos o más	10 (10,3)	36 (13,5)		Ref.^‡‡^	0,78 (0,36-1,68)
Conoce la inserción en la UBS^¶^ (n**=321)			0,334		
Sí	14 (16,9)	52 (21,9)			
Intento de inserción en la UBS^¶^ (n**=321)			0,485		
Sí	26 (31,3)	65 (27,3)		Ref.^‡‡^	0,82 (0,48-1,42)
Fuente de información sobre DIU^§§^ (n**=363)			0,619		
Médico	21 (7,9)	6 (6,2)			
Enfermero	20 (7,5)	10 (10,3)		Ref.^‡‡^	1,75 (0,54-5,71)
Otros^||||^	225 (84,6)	81 (83,5)		Ref.^‡‡^	1,26 (0,49-3,23)
Tiempo previsto para la inserción del DIU^§§^ (en meses) (n**=363)			0,232		
<1	13 (13,4)	33 (12,4)			
1-3	52 (53,6)	153 (57,5)			
4-6	22 (22,7)	39 (14,1)			
7 o más	10 (19,6)	41 (80,4)			
Número de intentos de inserción del DIU^§§^ (n**=361)			0,972		
Ninguno	42 (43,7)	114 (43,1)			
Uno	38 (39,6)	104 (39,2)			
Dos o más	16 (16,7)	47 (17,7)			
Pensó en rendirse (n**=363)			0,124		
Sí	25 (25,8)	49 (18,4)		Ref.^‡‡^	1,54 (0,89-2,67)

*RAS = Red de Atención a la Salud; ^†^n (%) = Frecuencia absoluta y porcentual; ^‡^Prueba de chi-cuadrado de Pearson; ^§^ORna = *Odds ratio* no ajustado; ^||^IC del 95% = Intervalo de confianza del 95%; ^¶^UBS = Unidad Básica de Salud; **n = Número total de personas que respondieron la pregunta; ^††^PR = Planificación reproductiva; ^‡‡^Ref. = Categoría de referencia; ^§§^DIU = Dispositivo intrauterino; ^||||^Amigo/Familiares/Portero, secretaria y profesora

**Tabla 4:** Proporción y *Odds Ratio* (OR) de uso de la RAS* por mujeres que insertaron el dispositivo intrauterino después de asistir al Ambulatorio de Planificación Reproductiva para acceder al método anticonceptivo en el servicio de referencia según la educación (n = 379). Belo Horizonte, MG, Brasil, 2024

Variables de Utilización de la RAS*	Educación (en años)		Valor p^‡^	Educación (en años)	
	0 a 12	13 o más		0 a 12	13 o más
	n (%)^†^	n (%)^†^		ORna^§^ (IC95%)^||^	
Conoce UBS^¶^ de referencia (n**=379)			0,282			
Sí	206 (89,6)	128 (86,0)			
Inscripción en la UBS^¶^ (n**=335)			0,333			
Sí	198 (96,1)	121 (93,8)			
Asistida en la UBS^¶^ en el último año (n**=311)			<0,0001^††^			
Sí	78 (65,6)	165 (85,9)		Ref.^‡‡^	3,21 (1,84-5,60)^§§^
Discutió PR^||||^ con profesional (n**=378)			0,073			
Sí	63 (42,3)	76 (33,2)		Ref.^‡‡^	0,68 (0,44-1,04)
Consulta con profesional para elegir el DIU^¶¶^ (n**=379)			0,021^††^			
Sim	66 (44,3)	75 (32,6)		Ref.^‡‡^	0,61 (0,40-0,93)^§§^
Profesional consultado (n**=113)			0,342			
Médico	24 (60,0)	37 (50,7)		Ref.^‡‡^	
Enfermero	16 (40,0)	36 (49,3)			1,46 (0,67-3,19)
Número de profesionales consultados (n**=379)			0,090			
Ninguno	66 (44,3)	128 (55,7)		Ref.^‡‡^	
Uno	59 (39,6)	75 (32,6)			0,66 (0,42-1,03)
Dos o más	24 (16,1)	27 (11,7)			0,58 (0,31-1,08)
Conoce la inserción en la UBS^¶^ (n**=335)			0,414			
Sí	24 (18,6)	46 (22,3)			
Intento de inserción en la UBS^¶^ (n**=335)			0,096			
Sí	31 (24,0)	67 (32,52)		Ref.^‡‡^	1,52 (0,93-2,51)
Fuente de información sobre DIU (n**=379)			0,783			
Médico	11 (7,4)	19 (8,3)		Ref.^‡‡^	
Enfermero	11 (7,4)	21 (9,1)			1,10 (0,39-3,13)
Otros***	127 (85,2)	190 (82,6)			0,87 (0,40-1,88)
Tiempo previsto para la inserción del DIU^¶¶^ (en meses) (n**=379)			0,015^††^			
<1	24 (16,1)	24 (10,4)		Ref.^‡‡^	
1-3	74 (49,7)	137 (59,6)			1,85 (0,98-3,48)
4-6	35 (23,5)	32 (13,9)			0,91 (0,44-1,92)
7 o más	16 (10,7)	37 (16,1)			2,31 (1,02-5,22)^§§^
Número de intentos de inserción del DIU^¶¶^ (n**=377)			0,043^††^			
Ninguno	61 (41,2)	101 (44,1)		Ref.^‡‡^	
Uno	68 (45,9)	80 (34,9)			0,71 (0,45-1,12)
Dos o más	19 (12,8)	48 (21,0)			1,56 (0,82-2,83)
Pensó en rendirse (n**=379)			0,988			
Sí	48 (28,9)	31 (20,8)			

*RAS = Red de Atención a la Salud; ^†^n (%) = Frecuencia absoluta y porcentual; ^‡^Prueba de chi-cuadrado de Pearson; ^§^ORna = *Odds ratio* no ajustado; ^||^IC del 95% = Intervalo de confianza del 95%; ^¶^UBS = Unidad Básica de Salud; **n = Número total de personas que respondieron la pregunta; ^††^Valor de p estadísticamente significativo (p<0,05); ^‡‡^Ref.= Categoría de referencia; ^§§^ *Odds ratios* con significación estadística, identificadas por el IC95% que no incluye el valor nulo (OR = 1); ^||||^PR = Planificación reproductiva; ^¶¶^DIU = Dispositivo intrauterino; ***Amigo/Familiares/Portero, secretaria y profesora

En cuanto al análisis del uso de la RAS según la paridad, según la Tabla 5, las multíparas tienen más probabilidades de conocer la UBS de referencia (OR=2,83; IC95% 1,48-5,42) y conocimiento sobre la inserción del DIU en la UBS (OR=3,14; IC95% 1,66-5,91) en comparación con las mujeres nulíparas. Sin embargo, estas mujeres tenían menos probabilidades de esperar de 1 a 3 meses para la inserción del DIU (OR = 0,42; IC95% 0,21-0,83) en comparación con las mujeres nulíparas. Además, tenían más probabilidades de tener un mayor número de intentos de inserción (2 o más intentos: OR= 2,15; IC95%: 1,17-3,96) y tuvieron menos probabilidades de abandonar (OR= 0,50; IC95% 0,30-0,82), en comparación con las mujeres nulíparas.

**Tabla 5:** Proporción y *Odds Ratio* (OR) de utilización de la RAS* por mujeres que insertaron el dispositivo intrauterino después de ser tratadas en el Ambulatorio de Planificación Reproductiva para acceder al método anticonceptivo en el servicio de referencia según paridad (n = 378). Belo Horizonte, MG, Brasil, 2024

Variables de Utilización de la RAS*	Paridad		Valor p^‡^	Paridad	
	Nulípara	Primípara/Multípara		Nulípara	Primípara/Multípara
	n (%)^†^	n (%)^†^		ORna^§^ (IC95%)^||^	
Conoce UBS^¶^ de referencia (n**=378)			0,001^††^		
Sí	130 (81,8)	203 (92,7)		Ref.^‡‡^	2,83 (1,48-5,42)^§§^
Inscripción en la UBS^¶^ (n**=334)			0,047^††^		
Sí	120 (92,3)	198 (97,1)		Ref.^‡‡^	2,75 (0,97-7,76)
Asistida en la UBS^¶^ en el último año (n**=310)			0,052		
Sí	86 (72,3)	156 (81,7)		Ref.^‡‡^	1,71 (0,99-2,95)
Discutió PR^||||^ con profesional (n**=377)			0,357		
Sí	54 (34,2)	85 (38,8)			
Consulta con profesional para eligir el DIU^¶¶^ (n**=378)			0,947		
Sí	59 (37,1)	82 (37,4)			
Profesional consultado (n**=113)			0,680		
Médico	21 (56,8)	40 (52,6)		Ref.^‡‡^	1,18 (0,54-2,60)
Enfermero	16 (43,2)	36 (47,4)			
Número de profesionales consultados (n**=378)			0,145		
Ninguno	88 (55,4)	106 (48,4)			0,86 (0,55-1,35)
Uno	56 (35,2)	78 (35,6)		Ref.^‡‡^	
Dos o más	15 (9,4)	35 (16,0)			1,68 (0,84-3,36)
Conoce la inserción en la UBS^¶^ (n**=334)			<0,0001^††^		
Sí	14 (10,8)	56 (27,5)		Ref.^‡‡^	3,14 (1,66-5,91)^§§^
Intento de inserción en la UBS^¶^ (n**=334)			0,003^††^		
Sí	26 (20,0)	72 (35,3)		Ref.^‡‡^	2,18 (1,3-3,65)^§§^
Fuente de información del DIU^¶¶^ (n**=378)			0,002^††^		
Médico	9 (5,7)	21 (9,6)		Ref.^‡‡^	2,31 (0,67-7,94)
Enfermero	5 (3,1)	27 (12,3)		Ref.^‡‡^	0,51 (0,22-1,13)
Otros***	145 (91,2)	171 (78,1)			
Tiempo previsto para la inserción del DIU^¶¶^ (en meses) (n**=378)			0,003^††^		
<1	14 (8,8)	33 (15,1)		Ref.^‡‡^	
1-3	106 (66,7)	105 (48,0)			0,42 (0,21-0,83)^§§^
4-6	24 (15,1)	43 (19,6)			0,76 (0,34-1,69)
7 o más	15 (9,4)	38 (17,3)			1,07 (0,45-2,55)
Número de intentos de inserción del DIU^¶¶^ (n**=376)			0,042^††^		
Ninguno	77 (48,7)	84 (38,5)		Ref.^‡‡^	
Uno	61 (38,6)	87 (39,9)			1,31 (0,83-2,05)
Dos o más	20 (12,7)	47 (21,6)			2,15 (1,17-3,96)^§§^
Pensó en rendirse (n**=378)			0,006^††^		
Sí	44 (27,7)	35 (16,0)		Ref.^‡‡^	0,50 (0,30-0,82)^§§^

*RAS = Red de Atención a la Salud; ^†^n (%) = Frecuencia absoluta y porcentual; ^‡^Prueba de chi-cuadrado de Pearson; ^§^ORna = *Odds ratio* no ajustado; ^||^IC del 95% = Intervalo de confianza del 95%; ^¶^UBS = Unidad Básica de Salud; **n = Número total de personas que respondieron la pregunta; ^††^Valor de p estadísticamente significativo (p<0,05); ^‡‡^Ref.= Categoría de referencia; ^§§^ *Odds ratios* con significación estadística, identificadas por el IC95% que no incluye el valor nulo (OR = 1); ^||||^PR = Planificación reproductiva; ^¶¶^DIU = Dispositivo intrauterino; ***Amigo/Familiares/Portero, secretaria y profesora

## Discusión

Los hallazgos del presente estudio indican que, a pesar de que la mayoría de las mujeres conocen y utilizan los servicios de la APS, cuando se trata del uso de servicios específicos para acceder al DIU, hay una reducción significativa en el acceso a la orientación sobre PR, sobre la posibilidad de insertar el DIU en este servicio y la falta de conocimiento de las mujeres sobre la posibilidad de insertar el DIU en la UBS. Además, esta reducción fue aún mayor para las mujeres con menor educación y nulíparas. Aún, hubo una baja proporción de derivaciones por parte de profesionales para la inserción del DIU en la consulta externa de referencia y una mayor proporción de mujeres que esperan más de un mes.

Este resultado confirma la hipótesis de este estudio, ya que señala barreras para la utilización de la RAS para el acceso al DIU, con una trayectoria asistencial marcada por la peregrinación de las mujeres a través de los servicios, fenómeno ya descrito en otras dimensiones de la atención a la salud, como el parto^37^ ^)^ y otros contextos de atención a la salud sexual y reproductiva^25^ ^,^ ^30^. También confirma la hipótesis de mayor dificultad para lograr la inserción del DIU en la RAS entre las mujeres en situación de mayor vulnerabilidad social, entre las que tienen menos educación y especialmente entre las que tienen menor paridad. Sin embargo, no se observaron desigualdades en el uso de los servicios de la RAS según la raza/color de piel.

En cuanto a las barreras para la utilización de la RAS para acceder al DIU en el municipio, las mujeres relataron dificultades aún no reportadas en estudios previos, como la ausencia de derivación al servicio de referencia, el desconocimiento de la posibilidad de inserción en la propia UBS y el tiempo de espera de entre 1 y 3 meses para la inserción. Estos hallazgos demuestran la dificultad de acceso y la fragilidad de los servicios de la RAS para garantizar los derechos sexuales y reproductivos en el contexto de la anticoncepción, barreras adicionales a lo relatado en estudios previos^2^ ^,^ ^11^ ^,^ ^25^.

La dificultad para acceder al DIU en la red de Belo Horizonte, evidenciada en el presente estudio, podría ser una realidad en otros grandes municipios brasileños, ya que más de la mitad de los municipios relataron no disponer del DIU en los servicios de la APS^11^. Además, la evidencia de estudios en otras localidades, incluyendo Minas Gerais, apunta al hecho de que la realización del procedimiento es restringida y no requiere la indicación de profesionales médicos^8^ ^-^ ^9^ ^,^ ^38^, muchas veces especialistas, que no están presentes en todos los servicios de APS, además del conocimiento inadecuado de los profesionales de la salud sobre el DIU^38^ ^-^ ^39^. Tales factores constituyen una barrera institucional que afecta la efectividad del derecho.

Esta realidad se agrava por el incumplimiento de los plazos legales de acceso al método, como se evidencia en este estudio, en el que la mayoría de las mujeres esperaron más de 30 días, no cumpliendo con lo establecido en la Ley de Planificación Reproductiva^40^, y aumenta el riesgo de embarazo no deseado. Este retraso puede estar relacionado con la disponibilidad del método en los servicios, ya que un estudio nacional que evaluó la disponibilidad de suministros en la APS para PR en 2012, 2014 y 2018 identificó, en todos los años, menor disponibilidad del DIU en estos servicios^11^. Este hecho también podría explicar la mayor falta de conocimiento relatada por las mujeres sobre la disponibilidad de DIU en las UBS encontrada en el presente estudio.

También en relación con las barreras, cabe destacar que la tasa de no inserción del DIU el día de la consulta también exige atención, aunque no se identificaron diferencias significativas entre los grupos en cuanto al uso de la RAS y los criterios clínicos. La imposibilidad de descartar el embarazo fue la principal razón para no insertar el DIU en el presente estudio y pudo ser superada con estrategias educativas, consejería reproductiva y flujos de atención adecuados^41^.

El análisis de los factores predisponentes y facilitadores, según lo previsto en el Modelo de Comportamiento para el Uso de los Servicios de Salud de Andersen^36^, evidenció desigualdades sociales en el uso de la RAS para la inserción del método. Se observó una relación entre la educación superior y el uso de los servicios de salud, corroborando los hallazgos de estudios internacionales^21^ ^,^ ^28^. Esta relación podría explicarse por el hecho de que los niveles más altos de educación contribuyen a transformar los patrones reproductivos femeninos al fortalecer la capacidad de toma de decisiones, ampliar el acceso a la información y favorecer la ascensión social^42^. Un estudio anterior también indicó un mayor conocimiento sobre el DIU de estas mujeres^23^. Además, la baja escolaridad podría dificultar el uso del DIU debido a la dificultad para comprender las orientaciones de los profesionales^43^.

Además de la educación, el estudio apunta a desigualdades relacionadas con la paridad, ya que las mujeres multíparas tenían mayor conocimiento y uso de la UBS para la inserción del DIU. Sin embargo, también esperaron y buscaron más activamente ser incluidas en el servicio que las mujeres nulíparas. La mejor situación de las multíparas podría explicarse por la mayor facilidad y oportunidades de acceso a los servicios de salud para las demandas de la línea de atención continua materno-infantil, que se extiende desde la atención prenatal hasta el seguimiento infantil^3^. Este hallazgo corrobora los resultados de estudios previos en el escenario internacional^44^ ^-^ ^45^. Además, se encontraron resultados similares en el escenario nacional^46^, pero abarcando todos los métodos anticonceptivos. Estos hallazgos demuestran una mayor apreciación de la reproducción, en la que las mujeres que son madres tienen un mayor acceso a los servicios.

 Así, las mujeres jóvenes y solteras, que constituyen la mayoría de las mujeres nulíparas, están excluidas del acceso y uso de los servicios, a pesar de que tienen demandas de anticoncepción. Este escenario puede, además de conducir a la desigualdad social, comprometer el pleno ejercicio de los derechos reproductivos y se configura como injusticia anticonceptiva^47^.

Un avance del presente estudio es haber analizado los datos de un servicio de referencia para la inserción del DIU con el trabajo de enfermeras obstétricas. Se sabe que el papel de los enfermeros es estratégico para ampliar el acceso a los DIU en la APS, especialmente debido a su inserción territorial en el ámbito del SUS. La directriz nacional^7^ ^)^ reconoce y recomienda el trabajo de estos profesionales para fortalecer la planificación reproductiva y familiar, considerando las necesidades de las personas a lo largo de las diferentes fases del ciclo de vida. Al atribuir a estos profesionales competencias para la inserción y prescripción del DIU, se refuerza su papel en garantizar el acceso al método, contribuyendo a la reducción de embarazos no planificados, abortos inseguros y morbilidad y mortalidad materno-infantil^48^.

Esta estrategia se ha implementado en algunas regiones del país, como São Paulo^49^, Florianópolis^50^, Mato Grosso do Sul^51^ ^)^ y varios municipios de la región Nordeste. Como resultado, los enfermeros de la APS fueron capacitados y realizan la inserción del DIU en estos municipios^14^. El desempeño consistente de los enfermeros en la inserción del DIU en estos lugares permitió ampliar el acceso al DIU, reduciendo el tiempo de espera para obtener el método, promoviendo el derecho a la elección libre e informada y contribuyendo a una mayor capacidad de resolución de problemas de la RAS. En otros países, como Australia^15^, Estados Unidos^16^ e India^17^, la práctica de los enfermeros está establecida y sistematizada en el acceso al DIU.

Además, un estudio sobre la calidad de la atención prestada a las enfermeras obstétricas en São Paulo mostró que más de 98% de las mujeres que insertaron DIU con parteras y enfermeras obstétricas relataron que recibieron orientación sobre PR antes de la inserción y que, de ellas, 98,6% consideraron suficiente para sentirse seguras con el uso del DIU^49^. Datos del Sistema de Información en Salud para la Atención Primaria (SISAB) indican que los enfermeros son responsables por la mayoría de las consultas individuales relacionadas con el DIU, representando alrededor de 76%^8^. Así, el papel de los enfermeros en la inserción del DIU amplía las oportunidades de las mujeres tanto para el conocimiento de los métodos LARC como para el acceso efectivo a su utilización^7^.

Sin embargo, en la APS de Belo Horizonte, los enfermeros no están incluidos en los protocolos locales de atención para la inserción y prescripción del DIU durante la consulta de enfermería, incluso con las recomendaciones de los reglamentos ministeriales^7^ y en la Resolución del Consejo Federal de Enfermería n.º 690/2022^14^. Este hecho podría contribuir a los resultados de este estudio, evidenciando barreras en la trayectoria de atención de las mujeres en los servicios de salud de la RAS para acceder al método, reforzando la existencia de problemas programáticos y ejecutivos en la provisión del método. Cabe destacar que el desempeño consistente de los enfermeros en la inserción del DIU permitiría ampliar el acceso a la anticoncepción reversible de acción prolongada, la reducción del tiempo de espera, además de una alternativa viable para llenar los vacíos de atención.

A pesar de los avances en el ámbito de las políticas públicas nacionales e internacionales en las políticas de salud de la mujer^6^ y de la ampliación del acceso a métodos anticonceptivos, incluido el DIU, en el ámbito del SUS, persisten desafíos, como la falta de información sobre los MC disponibles y la capacidad de los servicios para ofrecerlos. Superar estos desafíos es esencial para garantizar la realización del derecho de las mujeres a la salud y el pleno ejercicio de sus derechos reproductivos en Brasil.

Este estudio tiene limitaciones, como la evaluación exclusiva de mujeres atendidas en un servicio de referencia para la inserción del DIU, lo que podría considerarse un sesgo de selección, ya que las que asisten a las consultas pueden tener una mayor motivación en relación al uso de MC y DIU. Por lo tanto, los resultados pueden no reflejar las experiencias de las mujeres que no buscaron estos servicios por diversas razones, como la falta de conocimiento, las dificultades financieras, geográficas y culturales. Por lo tanto, es posible que la coordinación de la RAS sea incluso peor que la identificada en el estudio, representando un sesgo conservador, ya que la dificultad de las mujeres para utilizar la RAS para acceder al método puede haber sido subestimada. Por lo tanto, la generalización de los resultados debe hacerse con precaución. 

Por otro lado, esta metodología nos permitió recuperar información sobre la ruta previa de estas usuarias hasta llegar al servicio de referencia para la inserción del DIU, incluyendo barreras previas en la RAS. Entrevistarlos de manera más completa a lo largo de la red de un municipio grande puede ser costoso y propenso a varias fallas, como se describe en estudios anteriores^52^. Otra limitación fue la capacidad de los datos para referirse al uso de los servicios de salud en la RAS, que es parte del acceso en el sentido más amplio, pero no está configurado como una medida de acceso per se. Sin embargo, el estudio avanza investigando el uso de servicios para lograr el método en una capital brasileña con una muestra representativa. 

Los hallazgos del presente estudio tienen implicaciones para la práctica profesional y la gestión de la salud, destacando la necesidad de fortalecer la articulación entre las macropolíticas nacionales y las acciones locales, más específicamente en relación con el acuerdo de la RAS y los servicios de referencia, y crear estrategias para que los servicios de salud superen la lógica materno-infantilista que orienta la oferta anticonceptiva condicionada a la maternidad, garantizar el acceso efectivo a todas las mujeres, independientemente de su historia gestacional. Además, es fundamental intensificar los esfuerzos para mejorar el conocimiento de las mujeres sobre la disponibilidad y accesibilidad de los servicios de Salud Sexual y Reproductiva en la RAS, revisión de los protocolos municipales de PR para que incluyan a las enfermeras en la prescripción, inserción, revisión y mantenimiento del DIU. Así, se ampliaría la oferta de servicios a las usuarias de la APS, asegurando la autonomía femenina en la libre elección informada y en la reducción de las desigualdades.

## Conclusión

Los hallazgos del presente estudio apuntan a nuevas barreras para el acceso al DIU. Además, señalan desigualdades sociales en el uso de la RAS del servicio en relación con la escolaridad y la paridad. Dicha evidencia refuerza la necesidad de políticas públicas que prioricen la equidad en el acceso a la anticoncepción y reconozcan el papel estratégico de las enfermeras en la garantía de los derechos sexuales y reproductivos.

## Declaración de Disponibilidad de Datos:

El conjunto de datos de este artículo está disponible en la página de la RLAE en el repositorio SciELO Data, en el enlace https://www.enf.ufmg.br/pos/tese.php
